# A sequence level model of an intact locus predicts the location and function of nonadditive enhancers

**DOI:** 10.1371/journal.pone.0180861

**Published:** 2017-07-17

**Authors:** Kenneth A. Barr, John Reinitz

**Affiliations:** 1 Committee on Genetics, Genomics, and Systems Biology, University of Chicago, Chicago, Illinois, United States of America; 2 Department of Statistics, University of Chicago, Chicago, Illinois, United States of America; 3 Department of Ecology and Evolution, University of Chicago, Chicago, Illinois, United States of America; 4 Department of Molecular Genetics and Cell Biology, University of Chicago, Chicago, Illinois, United States of America; 5 Institute for Genomics and Systems Biology, University of Chicago, Chicago, Illinois, United States of America; Princeton University, UNITED STATES

## Abstract

Metazoan gene expression is controlled through the action of long stretches of noncoding DNA that contain enhancers—shorter sequences responsible for controlling a single aspect of a gene’s expression pattern. Models built on thermodynamics have shown how enhancers interpret protein concentration in order to determine specific levels of gene expression, but the emergent regulatory logic of a complete regulatory locus shows qualitative and quantitative differences from isolated enhancers. Such differences may arise from steric competition limiting the quantity of DNA that can simultaneously influence the transcription machinery. We incorporated this competition into a mechanistic model of gene regulation, generated efficient algorithms for this computation, and applied it to the regulation of *Drosophila even-skipped* (*eve*). This model finds the location of enhancers and identifies which factors control the boundaries of *eve* expression. This model predicts a new enhancer that, when assayed *in vivo*, drives expression in a non-*eve* pattern. Incorporation of chromatin accessibility eliminates this inconsistency.

## Introduction

Understanding how genetic function arises from the structural properties of genes is a fundamental problem of molecular genetics. With respect to the non-coding portions of genes, in prokaryotes there is a clear relationship between chemical properties and genetic function. In the *lac* operon, for example, there is a one-to-one mapping between the functional genetic unit of the operator and the structural/chemical unit of the binding site for *lac* repressor [1]. This level of understanding is absent in metazoan genes. The expression of many such genes is under the control of *cis*-acting DNA sequence which can span tens [2] to hundreds of thousands [3] of nucleotides. The central feature of such genes is the presence of enhancers, also known as *cis*-regulatory modules (CRMs). These sequences, which typically span 500 to 1000 base pairs (bp), recruit sequence-specific transcription factors to drive a subset of a gene’s full expression pattern [4–8]. Although enhancers are ubiquitous, how they arise from the underlying structure of genes remains obscure.

In this paper we address this problem by showing that under very general assumptions about underlying chemical mechanisms, the physical limitation that only a subset of distally bound transcription factors (TFs) can interact with the basal promoter complex at the same time induces a modular structure on a genetic locus. We consider a well characterized locus in *Drosophila melanogaster* known as *even-skipped* (*eve*). The enhancer structure of this gene has been exceptionally well characterizeed experimentally [4–7], and quantitative chemical models of the function of these enhancers are now well known [9–15].

The use of theoretical models in these studies is required because of the complexity of the chemical mechanisms underlying gene regulation. Experimental assays permit the dissection of a gene into its constituent parts and allow the properties of these parts to characterized in isolation. Models allow us to assay whether or not well defined interactions of these components give rise to the observed behavior of the intact system, and thus provide a minimal set of mechanisms required for understanding the biological phenomenon at hand. Theoretical models of whole loci have been constructed by assuming an underlying modular structure of enhancers and reconstructing the whole locus expression pattern from a weighted sum of of outputs of individual enhancers [16], but this does not address how this modular structure arises from underlying chemical interactions. Moreover, the fact that most developmental genes contain shadow enhancers [17–20] that behave nonadditively [21, 22] suggests that important regulatory mechanisms exist at the level of an intact locus that are not seen in isolated enhancers.

In this work, we construct a quantitative theory to explore the consequences of steric limitations on the amount of transcription factors that can simultaneously interact with proximal transcription complex. This form of enhancer competition is added to a previously existing model of gene regulation [14, 15, 23]. When applied to the *Drosophila eve* locus, which drives seven transverse stripes across the syncytial blastoderm, the model is able to fit the expression pattern and discover the factors that form each stripe. Furthermore, the underlying enhancer structure of the locus emerges from the internal structure of the model when it is fit to data, without such structure being imposed in the assumptions of the model. The model also shows the importance of chromatin accessibility in driving gene expression. Without consideration of such accessibility, the model predicts a new enhancer in the *eve* locus that, when assayed *in vivo*, drives expression in a non-*eve* pattern. Chromatin accessibility assays suggest that this fragment is inaccessible *in vivo* [24, 25]. A model that incorporates this accessibility data does not predict expression driven by this fragment within the intact locus.

## Materials and methods

### Model data inputs

Quantitative levels of *eve* mRNA along the AP axis have previously been reported for three lines in Drosophila Genetic Reference Panel (DGRP) [26] [27]. Data from L1 (RAL-437) at time class T6 was used for this study and is reported in S1 File. This data corresponded well to RNA data collected in 3D from CantonS [28]. Quanititative enhancer-reporter data was obtained from Staller *et al.* [29]. Relative transcription levels along the AP axis were obtained from the FlyEx database [30–33]. PWMs were derived from SELEX for factors Bcd, Hb, Kr, and Gt [34], bacterial-one hybrid for Kni and Cad [35], and footprinted sites for Tll [36] and Dst (http://line.bioinfolab.net/webgate/help/dxp.htm#D-stat-223). These PWMs have been used in prior work [14, 15].

### Sequence selection

The *eve* locus was taken from the *D. mel* assembly dm3 using coordinates 2R:5862089-5875238. A fragment spanning these bases was reported to drive the early nuclear cycle 14 seven stripe pattern [2, 4]. Multiple enhancers have been reported for *eve* stripes 2 and 3. For generation of S1 Fig we selected the enhancer with the greatest length for testing, as the longer enhancer is more likely to contain all DNA which drives a particular stripe. For this figure, the stripe 2 enhancer, sometimes called S2E, is the 800bp sequence spanning conserved blocks A and B reported in [37] and has dm3 coordinates 2R:5865217-5866014. The stripe 3+7 enhancer is the restriction fragment identified in [4] and has dm3 coordinates 2R:5863006-5863888. Both the stripe 4+6 enhancer and the stripe 5 enhancer were identified in [5]. These have respective dm3 coordinates of 2R:5871404-5872203 and 2R:5874230-5875033.

For the remainder of the work we used the sequences reported in Staller *et al.* [29] as these sequences can be directly compared quantitative to enhancer-reporter data from the same work.

### Data registration

All data in this work was registered against the transcription factor levels available in the FlyEx database. The *eve* RNA was imaged together with Eve protein so that the protein channel could be used for data registration. In order to use data from Staller *et al.* [29] nuclei in a 10% DV strip along AP axis were registered to the FlyEx database using the *eve* RNA channel. All data was registered using the BREReA [38, 39] software. We used the mean levels at each percent embryo length for comparison to predicted reporter expression.

### Parameter estimation

The model equations Eqs S1-S17 (in S1 Appendix), Eqs (1), (2) and (3) were implemented in C++ code. Optimization of model parameters was performed by minimizing the sum of squared differences (SSE) between the model and data using Lam-Delosme Simulated Annealing in serial [40–42]. Annealing parameters are given in S2 File. Below we describe the search space and controls for accuracy and significance.

#### Search space

The search space for each parameter was explicitly set (S2 File) in terms of a range for each parameter. These ranges were set to ensure that biologically relevant parameter values could be achieved. A TF, *a*, will have 3 to 5 associated parameters depending on its biological function. The first of these, *A*_*a*_ (S3 in S1 Appendix), converts activities observed as fluorescence and binding free energies obtained as PWM scores into chemical units. Intracellular activities of proteins range from about 1 to 1000 nM, and allowing for similar uncertainty in the affinities *K*, which always occur as products with *v* in the model equations (see S6 in S1 Appendix), we allow *A*_*a*_ a range of somewhat more than 6 orders of magnitude, from 10^−6^ to 4 × 10^0^. The next parameter, *λ*, scales differences in binding score to differences in relative affinity. Originally, values of 0.5 to 2 were proposed as a reasonable range for this parameter [43], but some PWMs used in this work were generated using multiple rounds of SELEX, which may under-represent low affinity binding sites. We extend the range of this parameter to be from 0.5 to 5 to allow for the possibility of over-specified PWMs. The range of the bicoid cooperativity *ω* was set to 1 to 1000. This corresponds to a Δ*G* of up to –7 kcal/mole, which is fully compatible with observed ranges for *λ* repressor and *Drosophila* TFs [44, 45].

The efficiency of transcription factors *E*^*Q*^ and *E*^*C*^ in repression or coactivation respectively always multiply the fractional occupancy *f*, and hence were fixed to their natural scale of 0 to 1. In contrast, the activation efficiency *E*^*A*^ also sets the scale of *N* and thus the steepness of promoter response to activation. We allowed *E*^*A*^ to vary from 0 to 25. At the high end of this range promoter response is sufficiently close to a step function that biologically undetectable changes in TF concentrations can switch the promoter between on and off states. *θ* ranged from 5 to 25 because it is subtracted from *N*, and values smaller than 5 allow for substantial transcription in absence of activation.

#### Optimization

Optimization was performed 20 times with *κ* [46] set to 1.6 × 10^−4^ and 80 times with *κ* set to 1.6 × 10^−5^, where smaller values of *κ* give more accurate results at the cost of additional computational time. Each optimization run was started from a random set of initial parameter values.

In order to verify that our optimization procedure is able to find the global minimum we require a scenario in which this global minimum is known. We construct such a test problem by replacing the data with the output of the parameter set reported in the main text of this work. In this case, there is a known global minimum at zero, where the learned parameters are the parameters of the fit used to construct the test problem. When we repeated this procedure 80 times, the best resulting parameter sets had scores several orders of magnitude lower than than those fit to data. The learned parameter sets were also well correlated with the parameter set used to generate the test problem. Spearmen *ρ* was 0.963, 0.952, and 0.934 for the best three fits respectively (Sheet “Fit Known Optimum” in S2 File). We obtained similar results when this control was repeated for models incorporating chromatin state (Sheet “Fit Known Optimum Chromatin” in S2 File).

The lowest scoring run (Model 12) in the initial set of 20 runs with *κ* = 1.6 × 10^−4^ was selected for the analysis in this work. We verified optimiztion accuracy by performing 80 additional runs with *κ* = 1.6 × 10^−5^ and subjecting the best three of these to further analysis. These runs had a 4% improvement in summed square error and gave parameter values that were well correlated with Model 12, having Spearman *ρ* of 0.969, 0.969, and 0.924 respectively (S2 File, sheet “Repeat”). These parameter sets did not differ significantly in their output or enhancer prediction (S8 Fig). We also repeated this procedure for the model incorporating chromatin state. Again, the best three fits had similar properties, owing to a very high correlation in the achieved parameter sets, which had Spearman *ρ* of 0.958, 0.958 and 0.969 respectively (Sheet “Repeat Chromatin” in S2 File). These did not differ significantly in their prediction of enhancer location or output (S9 Fig).

Overfitting is generally a concern when the number of parameters exceeds the number of data points. Here we are fitting 32 free parameters to 58 data points. Additionally, to confirm that this model was not overfit we tested whether permuted data could be used to drive the expression pattern. We permuted the non-coding sequence data and the best fits to this data had scores that were three times worse than the best fits to the *eve* locus (S2 File). None of these fits drove all of the six *eve* stripes that were in the modeled region.

### Calculation of contribution to stripe borders

At every AP position, the marginal contribution to transcription rate with respect to a change in each transcription factor concentration was calculated numerically by adding and subtracting from the concentration of each factor and calculating the predicted change in transcription rate while keeping all other parameters constant. Specifically, we estimate the quantity $\frac{\partial R_{i}}{\partial {\left[A\right]}_{i}}$ using numerical differentiation, where *R*_*i*_ is the predicted transcription rate at AP position *i* and [*A*]_*i*_ is the concentration of factor A at the same position. Where [*A*] is greater than 0 we use a symmetric difference quotient $\frac{f(x+h)-f(x-h)}{2h}$, otherwise we use Newton’s difference quotient $\frac{f(x+h)-f(x)}{h}$. We used changes in concentration of orders of magnitude 10^1^ to 10^−11^ to verify convergence of this estimate (S7 Fig). To calculate the contribution of each transcription factor to a change in transcription rate between adjacent AP positions, we multiply $\frac{\partial R_{i}}{\partial {\left[A\right]}_{i}}$ by the amount that the transcription factor is changing at that position △[*A*]_*i*_, given by $\frac{{\left[A\right]}_{i-1}+{\left[A\right]}_{i+1}}{2}$.

### Calculation of contribution to activation

To calculate the contribution of each factor towards the total transcription rate we first calculate the number of transcriptional adaptors recruited to each sequence by each factor $N_{i[m,m+\alpha ;a]}=\sum_{k:a_{i}=a_{k}}F_{k}E_{a_{k}}^{A}I\left(k,m,m+\alpha \right)$. Next, we find the number of transcriptional adaptors recruited to the TSS by each factor by taking time weighted sum *N*_*a*_ = ∑_*i*_*N*_*i*[*m*, *m*+*α*;*a*]_*T*_*i*_. We report the percent of adaptors recruited to the TSS by each factor 100(*N*_*a*_/max(*N*_*a*_)).

### Generation of reporter constructs

Reporter constructs where generated using a pCaSpeR backbone (GeneBank X81644.1) containing the promoter and first 22 amino acids of *eve* fused to *Lac*Z, generated by Small et al. [47]. An attB sequence was inserted into the multiple cloning site using the restriction enzyme Xba1 for insertion in the AttP2 landing site on chromosome 3 [48]. The enhancer sequence was extended by PCR primers containing overlap with this vector (S1 Appendix). The vector was then digested by enzymes EcoR1 and Xho1 and the enhancer was inserted using Gibson assembly [49]. The resulting vector was injected into flies of the genotype P{nos-phiC31\int.NLS}X, P{CaryP}attP2 by Rainbow Transgenics. Quantitative data was collected from these lines as previously described [50].

### Identification of accessible chromatin

Accessible chromatin regions defined by FAIRE-seq data by McKay 2013 [25] were obtained from GEO accession number GSE38727. Accessible chromatin regions defined by DNAse-seq data were obtained from Li 2011 [24] and were translated to dm3 coordinates using the UCSC genome browser LiftOver tool. Open chromatin regions were defined as the union of the two datasets.

## Results

### Sequence level model without enhancer competition and *eve* expression

In previous work, we generated a model of gene regulation that computes transcription rate from DNA sequence, transcription factor concentrations, and DNA-binding preferences in the form of position weight matrices (PWMs) [9, 14, 15, 23, 51]. In this model, we first calculate equilibrium transcription factor occupancy using thermodynamics. This calculation incorporates cooperative binding and repression through steric competition for binding. Second, we calculate context dependent switching between repressing and activating states, known as coactivation, wherein proteins activate only when bound in proximity to a bound coactivator. Third, we calculate the repressive effects of short-range quenching. Fourth, we calculate the number of transcriptional adaptors, proteins which interact with both DNA-bound transcription factors (TFs) and the transcription machinery [8], recruited to an enhancer by a weighted sum that represents the efficiency of adaptor recruitment for each activator. Finally, we treat these adaptors as catalysts that reduce the energy barrier to transcription and describe this in the form an a diffusion-limited Arrhenius rate law. For a complete description of these mechanisms, see S1 Appendix.

To test the ability of this model to describe the regulation of an entire locus, we applied this model to the *even-skipped* (*eve*) gene of *Drosophila melanogaster*. Confocal microscopy in *melanogaster* embryos has allowed quantification of both transcription factor levels and mRNA levels at single nucleus resolution along the anterior-posterior axis [31, 32]. These data amount to a set of quantitative single cell assays of transcription input and output in a native tissue context, providing an extraordinarily precise testbed for theoretical models.

We attempted to model the whole locus behavior of *eve* by two methods. First, we trained the above model on levels of *eve* mRNA from 35.5% to 92.5% embryo length, encompassing stripes 2 through 7, driven by 13,150 bp of *eve* DNA extending from 4730bp upstream to 8420 bp downstream of the transcription start site (TSS). This DNA is sufficient to drive the early seven stripe pattern [2, 4]. The model was able to drive the desired pattern from the regulatory sequence used (Panel A in S1 Fig), but when the parameters learned from this fit were confronted with smaller segments of sequence corresponding to the enhancers for each of the six stripes, none of the enhancers were predicted to drive expression (Panels B-E in S1 Fig). Similarly, when we trained the model simultaneously on each individual enhancer driving its respective stripe pattern, we are able to achieve good fits (Panels F-I in S1 Fig), but the parameters obtained predict that the intact locus will drive saturating expression across the entire embryo (Panel J in S1 Fig). No fits were able to simultaneously describe the the action of both the intact locus and individual enhancers, leading us to conclude that at least one additional regulatory mechanism emerges at the level the intact locus and is necessary to model its behavior.

### An enhancer competition model

One potential issue is the implicit assumption that factors bound to the entirety of modeled DNA simultaneously influence a promoter. Instead, only a finite length of *α* bp of DNA can simultaneously influence a gene’s promoter within a short timespan. We expect activators bound to DNA on scales smaller than this length to synergistically activate transcription through cooperative action on the basal transcription machinery, while activators separated by larger scales will compete for promoter occupancy. There has been much focus on “minimal” enhancers—the smallest segments that are able to recapitulate a pattern *in vivo*—but much larger sequences may be able to influence a promoter. Indeed, we find that when the 480 bp minimal stripe 2 element of *eve* (MSE2) is extended by 320 bp there is a five fold increase in transcription rate (S2 Fig). This suggests that sequences of up to *α* = 1 kb are able to simultaneously influence a promoter, and we use this value for the rest of this work. However, the final results were completely insensitive to setting *α* = 500 (S3 Fig and S2 File).

While we expect activators bound within a region smaller than 1kb to synergistically activate transcription, how disparate elements compete for access to a promoter is currently unknown. Recently, it has been shown that transcription driven by *Drosophila* developmental enhancers occurs in bursts [52] and that forced enhancer-promoter looping in murine cell lines indicates that the frequency of bursts is determined by the frequency of interaction with a promoter [53]. Collectively, this demonstrates that transcription rates can be controlled at the level of burst size or burst frequency and these quantities correspond to the rate of transcription induced by enhancer-promoter interactions and the frequency of such interactions respectively. For tested *Drosophila* enhancers, these quantities are highly correlated [54]. Thus, we propose that the frequency of enhancer-promoter interaction and the rate induced by such interaction is proportional to the number of transcriptional activators bound to a DNA segment.

Specifically, we imagine that, for any DNA segment bounded by base pairs [*m*, *m* + *α*], where *α*, introduced above, represents the length of DNA that simultaneously influence the promoter (the “window size”), that *N*_[*m*, *m* + *α*]_ transcription adaptors are recruited (For calculation of *N*, see [14] and S1 Appendix). The rate of mRNA synthesis, *R*_[*m*,*m* + *α*]_, driven when the segment interacts with the promoter is given by a diffusion-limited Arrhenius rate law

$$\begin{array}{c} R_{\left[m,m+\alpha \right]}=\frac{R_{\max}}{1+\exp(\theta -N_{\left[m,m+\alpha \right]})}, \end{array}$$ (1)

where we assume without loss of generality that a single bound coactivator lowers the Arrhenius energy barrier, Δ*A*, to transcription initiation by one unit. The free parameter *θ* is the total energy barrier which sets the rate of transcription in the absence of activation. The scale of both *N* and *θ* are effectively set by the fit to data.

For a locus of length *l*, the fraction of time that any DNA segment [*m*, *m* + *α*] influences the promoter is given by

$$\begin{array}{c} T_{\left[m,m+\alpha \right]}=\frac{\beta N_{\left[m,m+\alpha \right]}}{1+\sum_{n=1-\alpha }^{l}\beta N_{\left[n,n+\alpha \right]}}, \end{array}$$ (2)

where the free parameter *β* determines how much individual bound adaptors increase the frequency of interaction with the promoter. Note that the summation in the denominator is taken over every base position in the locus. The total rate of transcription driven by the locus is then given by the frequency-weighted sum of transcription due to each DNA segment [*m*, *m* + *α*], so that

$$\begin{array}{c} R_{\text{total}}=\sum_{m=1-\alpha }^{l}R_{\left[m,m+\alpha \right]}T_{\left[m,m+\alpha \right]}. \end{array}$$ (3)

Again, the summation occurs over all possible *α* subsequences of the *eve* locus iterated in single nucleotide increments. The half life of *lac*Z and *eve* mRNA is short compared to the timescale of changes in gene expression, so that

$$\begin{array}{c} \frac{d[\text{mRNA}]}{dt}\propto \left[\text{mRNA}\right], \end{array}$$ (4)

an observable quantity.

The calculation of transcription factor occupancy with full thermodynamics, which is used to calculate *N* (See S1 Appendix), requires enumeration of all possible binding states. In previous work this was done using an explicit calculation on each configuration [14]. Such a calculation scales with 2^*n*^ where *n* is the number of binding sites on a sequence. When performing calculations on the entire locus, we identified 2920 binding sites with a log-odds score greater than 0—the threshold used for calling binding sites in this work. Explicit calculation of 2^2920^ states is computationally infeasible. For this work we developed a new algorithm that uses dynamic programming. This new algorithm scales linearly with the number of binding sites and can efficiently calculate transcription factor occupancy at a genomic scale. A full description of this algorithm is included in S1 Appendix.

### Enhancer competition and *eve* expression

We trained the free parameters in the model given by Eqs S1-S17 (in S1 Appendix), Eqs (1), (2) and (3) to the expression of the *eve* locus from 35.5% to 92.5% embryo length, using the 13kb sequence described previously. We omitted stripe 1 from this study because its anterior border is controlled by transcription factors for which we do not have data. Additionally, anterior of stripe 1 the clean functional distinction between AP and dorsal-ventral (DV) patterning breaks down, and data along a single axis is inadequate.

**Fig 1:**
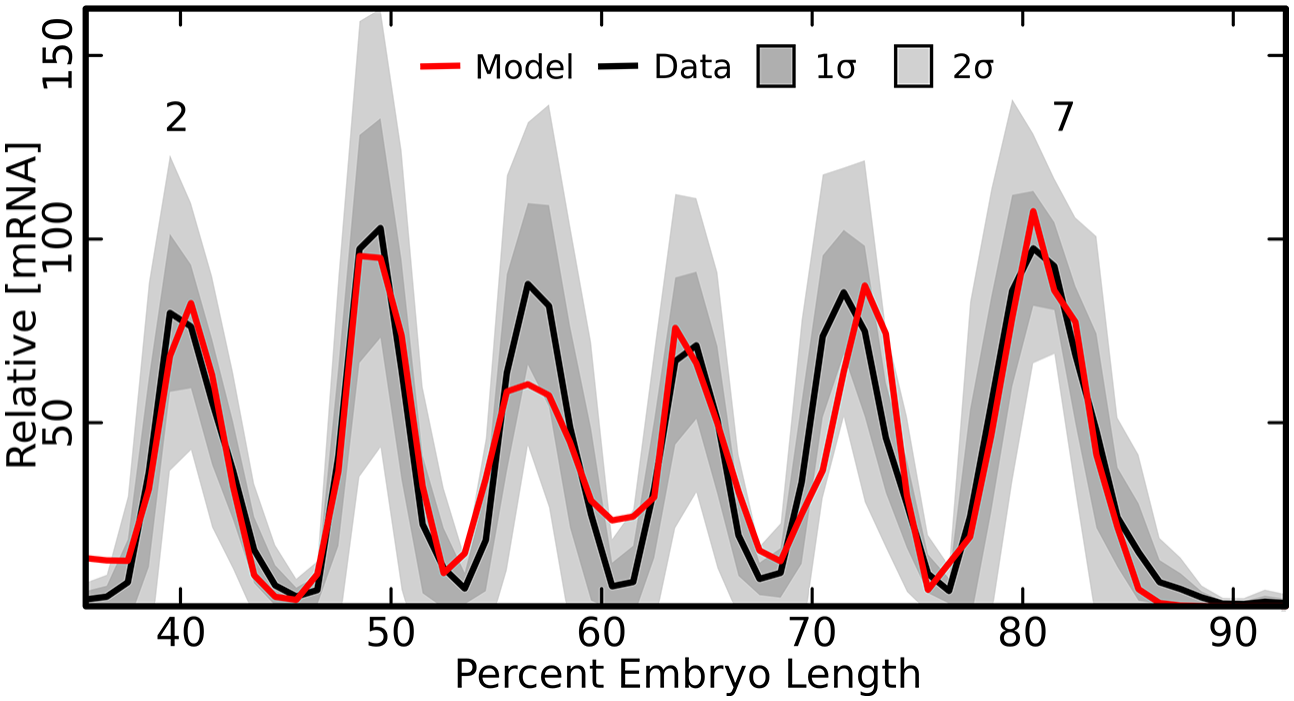
Model with enhancer competition trained on the *eve* locus. Observed mRNA levels (black line) are shown together with model output (red line). One (dark grey shading) and two (light grey shading) standard deviations about the mean of the data are shown. Data comes from 7 embryos for a total of 19 to 30 nuclei per embryo position. The axes are labeled; percent egg length is measured from the anterior pole.

The model was able to achieve a good fit to the expression pattern of *eve* stripes 2-7 (Fig 1). Specifically, the model was within two standard deviations of the data everywhere except at the 1-2 and 4-5 interstripes, and within one standard deviation of the data except at the two locations mentioned as well as at the peak of stripe 4, which is smaller than the data, and the margins of stripe 6, where the model produces a stripe displaced one nucleus to the posterior. Interestingly, the lag in the position of stripe 6 is consistent with the lag observed from the stripe 4+6 enhancer [29] indicating there may be reasons for the discrepancy between the enhancer and locus that are outside the scope of this model.

### Identification of *eve* enhancers

**Fig 2:**
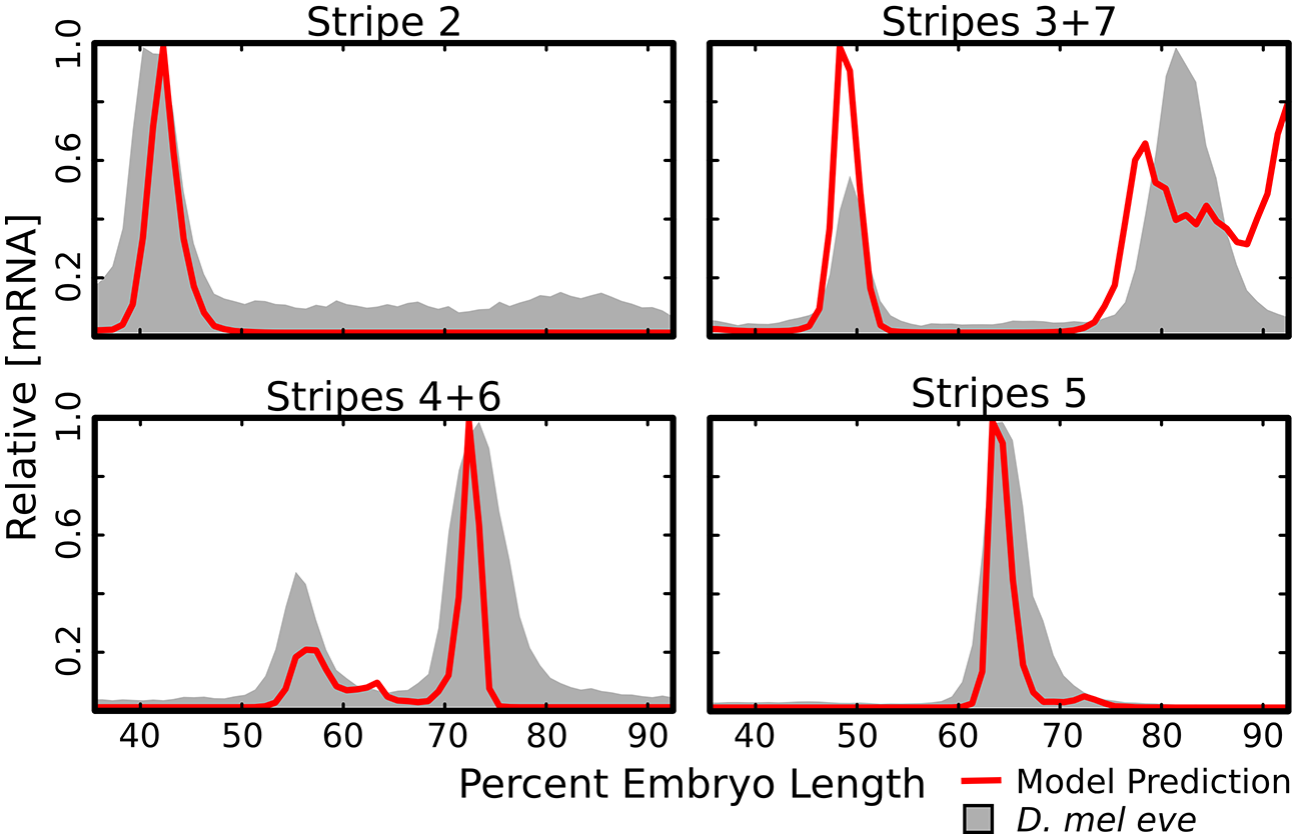
Predicted output of known *eve* enhancers. The trained model was used to predict the the transcription rate driven by four previously reported enhancers of *eve*. For each enhancer the predicted output was standardized such that 1 represents the maximum rate driven by that enhancer (red lines). The relative mRNA driven by each enhancer (gray shading) was obtained from Staller *et al.* [29]. This data, also standardized, is included for visual orientation within the embryo and levels are not commensurate with predicted enhancer output.

The *de novo* identification of enhancer locations and activity is a major goal of gene regulatory models. We tested the ability of the model to identify known enhancers in two ways. First, we used the trained model to simulate the activity of known enhancers of *eve* *in silico* (Fig 2) and compared this to quantitative data on the expression driven by each enhancer [29]. Each is correctly predicted to drive expression of its corresponding stripes. Quantitatively, there are some discrepancies. For the enhancer of stripe 3+7 we predict reduced output from stripe 7, which is consistent with the initial reports on this enhancer [7], but not with quantitative data. We predict poor anterior repression of stripe 7 when driven by the 3+7 enhancer. Additionally, we observe weak expression from stripe 4 when driven by the 4+6 enhancer. Generally, the predicted expression patterns are narrower than observed patterns.

**Fig 3:**
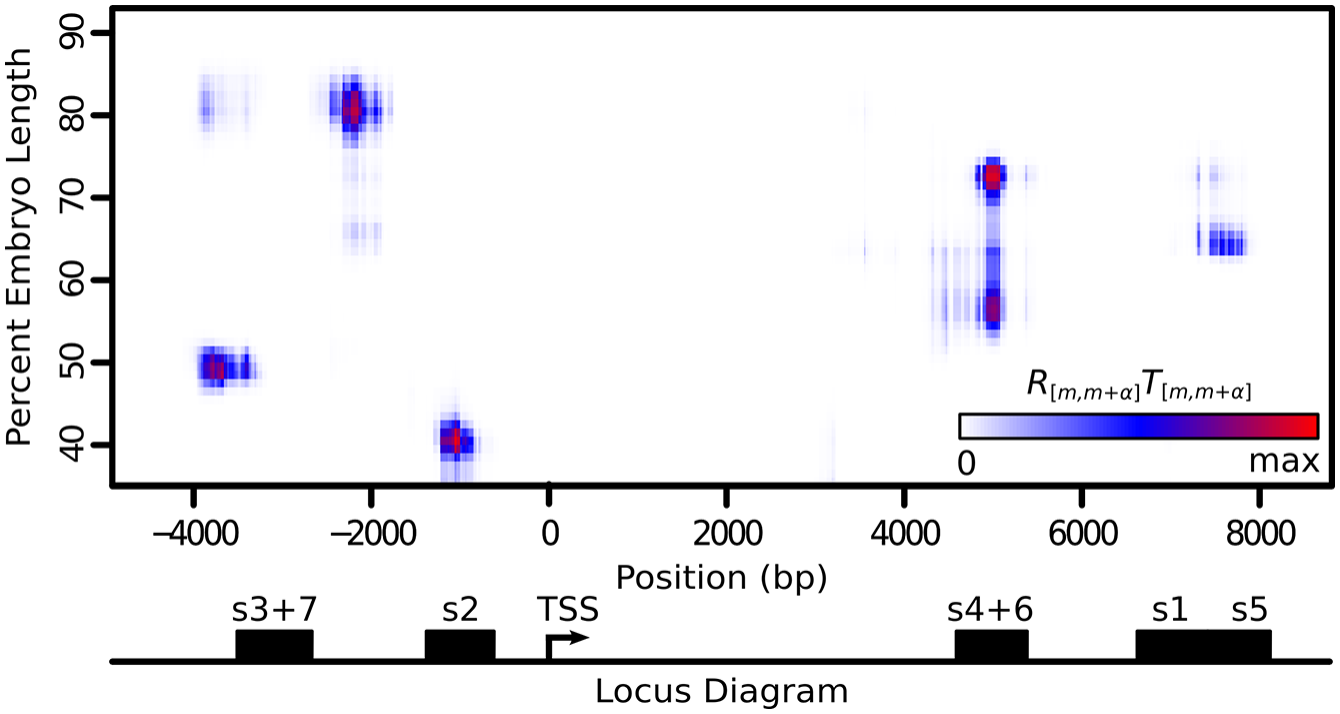
Expression contribution over space sequence and embryo length. We report a heat map of the quantity *R*_[*m*,*m*+*α*]_*T*_[*m*,*m*+*α*]_ (Eq (3)), which represents the amount each 1kb sequence, centered on base *m* + *α*/2 (*x*-axis), contributes towards total expression at each position in the embryo (y-axis). The color scale is standardized to the range of the data. The x-axis is labeled with a map of the *eve* locus, displaying the transcription start site and locations of previously identified enhancers (black rectangles).

Similarly, we looked at expression contributions across the entire *eve* locus by looking at the rate driven by every individual 1kb subsequence (Fig 3). We find that for stripes 2 through 6, the majority of activation is result of tightly clustered groups of sequences that have high overlap with the locations of previously reported enhancers. While stripes 2 through 6 have single clusters that drive their expression, we find that stripe 7 is driven not only by the stripe 3+7 enhancer, but also by DNA that lies 5’ of the stripe 2 enhancer. Expression driven by parts of this region have previously been reported for constructs that contain varying lengths of DNA 5’ of the stripe 2 enhancer [9, 29] and explains why deletions of the stripe 3+7 enhancer lead to loss of stripe 3, but not stripe 7 [4].

### Control of *eve* stripe domains

Three lines of evidence have been used to establish which factors control the boundaries of *eve* expression domains—mutations in *trans*, mutations in *cis*, and regulatory models—carried out in either the intact locus or enhancer-reporter constructs. In the best cases there is agreement between these techniques, for example in Giant (Gt) null embryos the anterior border of stripe 2 expands when driven by both native *eve* [55] and by a reporter for the proximal 2.9kb of the locus [56]. Similarly, there is a stripe 2 expansion when Gt binding sites were removed from reporters for either the proximal 5.2kb of *eve* [57] or MSE2 [6]. Collectively, these experiments provide strong evidence that Gt is responsible for forming the anterior boundary of *eve* stripe 2. Additionally, models of gene regulation have identified Gt as a key regulator of stripe 2 in both the locus [16, 40] and enhancers [9, 15].

For other *eve* borders there is conflicting evidence. For instance, in Kruppel (Kr) null embryos [55] the posterior of native *eve* stripe 2 expands, but the domain driven by MSE2 does not [6] indicating that other factors may contribute to this stripe border. Similarly, in Knirps (Kni) null embryos or after deletion of Kni sites, the minimal stripe 3 enhancer (MSE3) does not form a posterior border [7, 58], however stripe 3 forms normally in the intact locus [4, 7, 55]. Finally, the anterior border of stripe 7 appears to be regulated by Kni [7, 58, 59] when stripe 7 expression is driven by MSE3, or by Gt when expression is driven by the whole locus [55] or by an *eve* 2+7 enhancer [9, 29].

**Fig 4:**
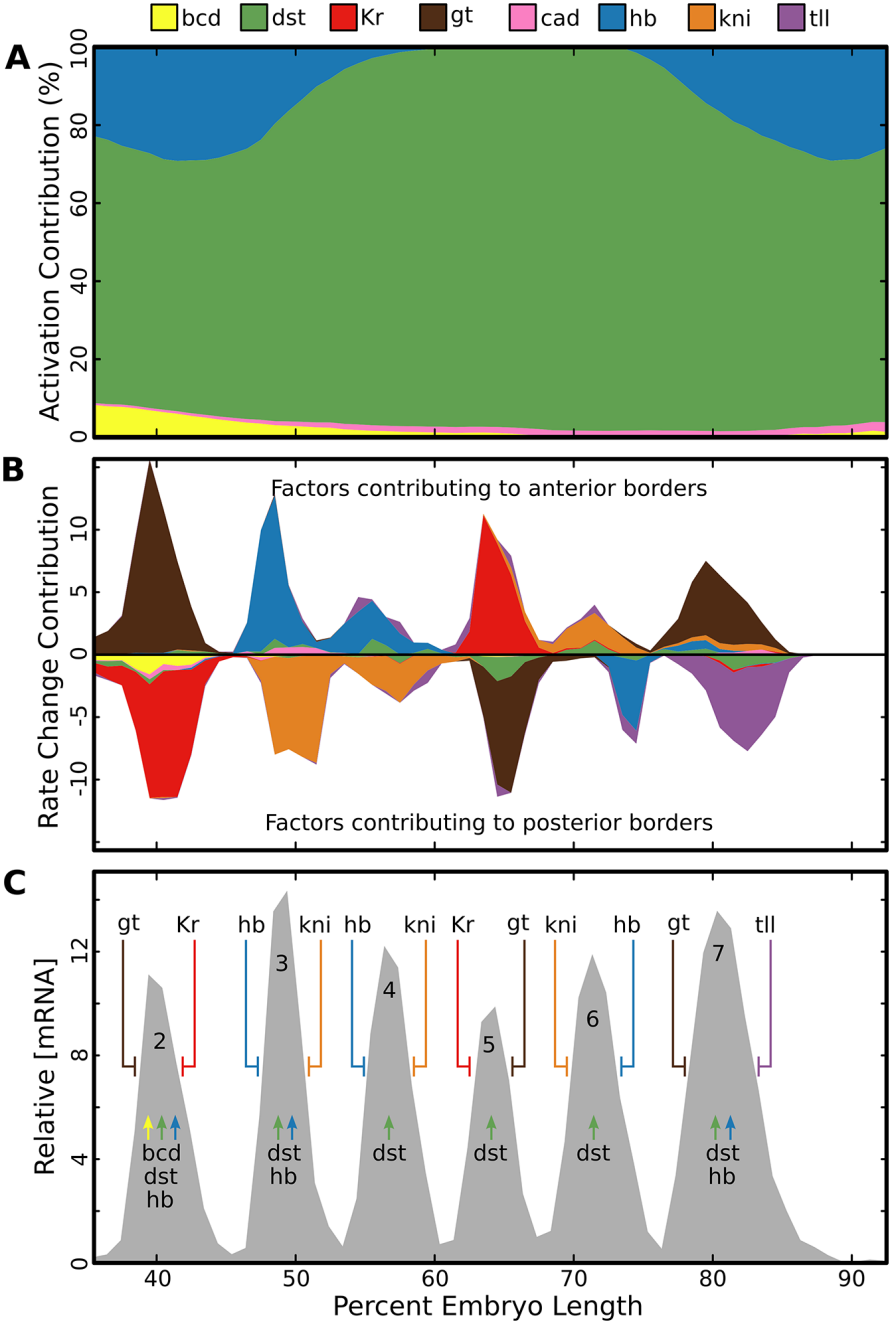
Mechanisms of activation and repression in the locus. A: Cumulative line graph showing the amount of *eve* mRNA attributable to each TF (y-axis) at each embryo position (x-axis). We calculated the percent of transcriptional adaptors *N* that are recruited by each TF to the transcription machinery at each embryo position (x-axis) and scaled total output by this value. For calculation, see Materials and Methods. B: Cumulative line graph showing the change in [mRNA] caused by a change in concentration of each TF (y-axis) at each embryo position (x-axis). The total sum gives the the change in [mRNA] at each embryo position. Thus, factors which contribute to anterior borders give positive values and those that contribute to posterior borders give negative values. For calculation, see Materials and Methods. C: A summary of the factors responsible for each expression feature of *eve* as determined by A and B. Activators are indicated by arrows and repressors by T-bars.

**Fig 5:**
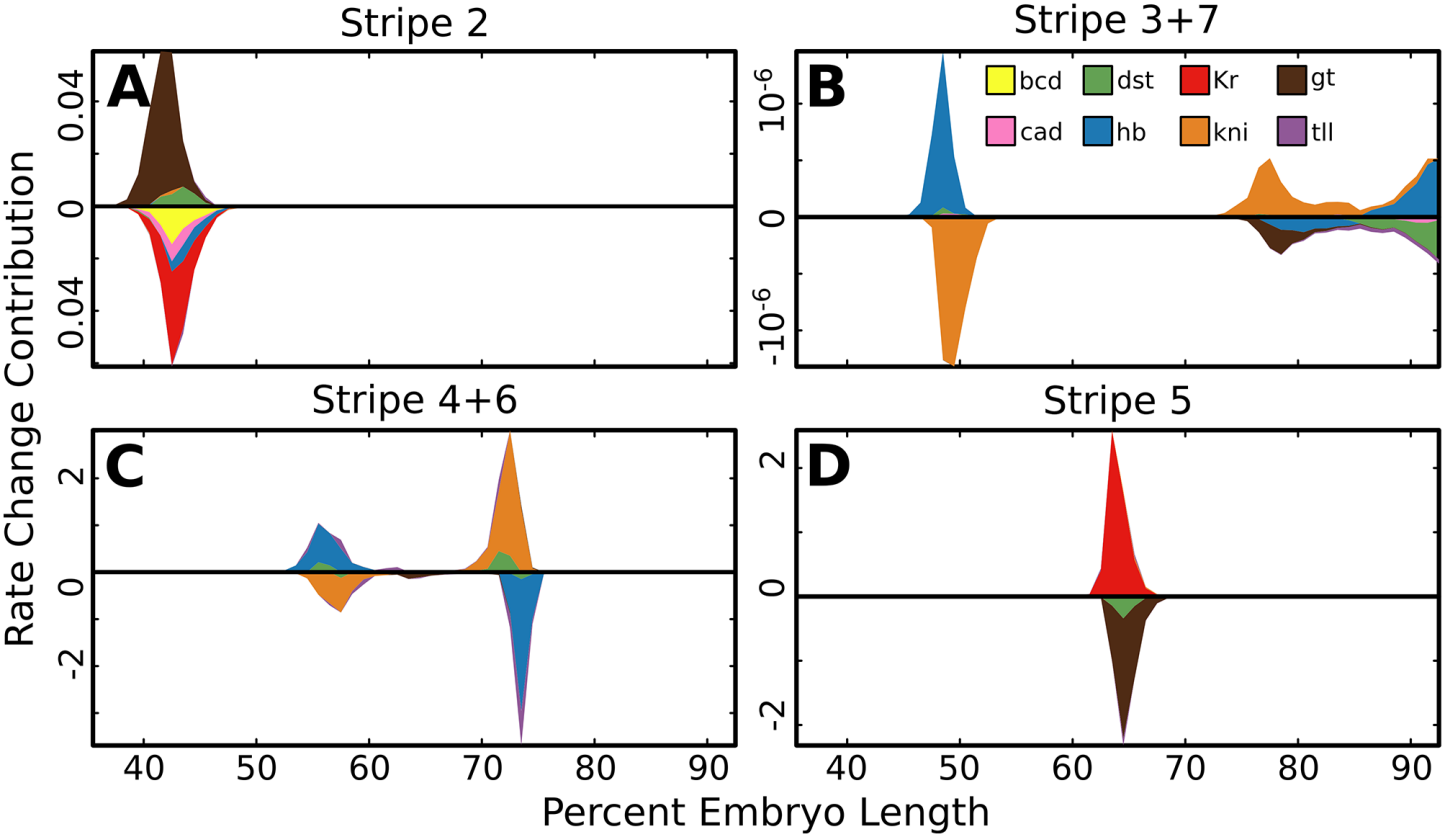
Mechanisms of repression in enhancers. For four previously reported *eve* enhancers the predicted contribution of each TF to a change in [mRNA], along the AP axis, was calculated as in Fig 4B and described in Materials and Methods.

In each of the above cases there are conflicting results from experiments where expression is driven by separate enhancers compared to those in which it is driven by the intact locus. In order to resolve these conflicts we identified the factors responsible for stripe boundaries in both the locus and individual enhancers in a single, unified, model. Given a trained set of model parameters, we are able to quantitatively decompose the change in [mRNA] in adjacent nuclei into the effects due to the changes in concentration of each transcription factor in both the locus (Fig 4B) and individual enhancers (Fig 5). Within the locus, in wild type *D. melanogaster*, we find that single transcriptional repressors are responsible for forming the boundaries of each stripe (Fig 4B, summarized in Fig 4C). For the factors forming the borders of stripes 4 through 6, the model identifies the same factors (Figs 4B, 5C and 5D) that have been previously identified through experiment [5, 59]. In agreement with previous literature [6, 40, 55–57], we find that Gt sets the anterior border of stripe 2 and that Kr defines the posterior boundary of that stripe in the intact locus. In contrast to the locus, we find that there is a significantly larger contribution from declining Bicoid (Bcd) and Hunchback (Hb) levels on MSE2 (Fig 5A), which potentially explains why expression driven by MSE2 does not shift to the posterior in Kr null embryos [6].

Next we examined the regulation of stripes 3 and 7. We find that in the intact locus, stripe 3 has anterior and posterior borders set respectively by Hb and Kni in both the intact locus (Fig 4B) and in the stripe 3+7 enhancer (Fig 5B). This result is consistent with previous reports [7, 58, 59], but falls short of explaining how stripe 3 forms in Kni mutants [7, 55]. We do not detect a contribution from Kr as suggested by a previous model [40]. For stripe 7 we find that Gt sets the anterior border in the intact locus, but we also find that that Kni sets this border when expression is driven by the stripe 3+7 enhancer. Similarly, we find that the posterior border of stripe 7 is primarily set by Tailless (Tll) repression when that stripe is driven by the whole locus. but that the posterior border of stripe 7, when driven by the 3+7 enhancer, is set by Hb.

**Fig 6:**
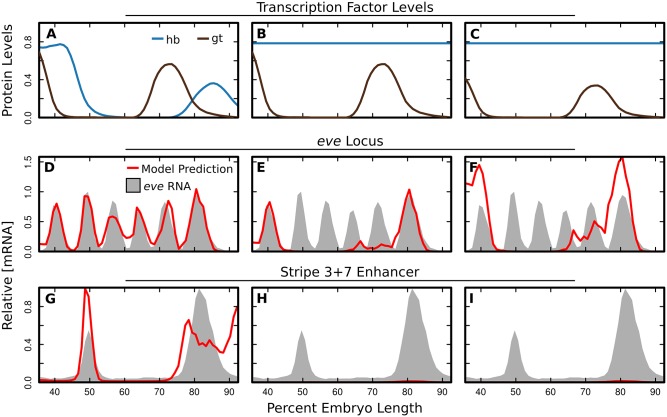
Predicted effects of ectopic Hb. A: The measured relative levels of Hb and Gt (y-axis) from 35.5% to 92.5% embryo length (x-axis) [30–33]. B: Simulated relative levels of Hb and Gt. Hb is set to a spatially uniform value and Gt is unchanged from A. C: Simulated relative levels of Hb and Gt. Hb is set to a spatially uniform value and Gt is reduced by 40%. D-F: Predicted relative [mRNA] levels (red lines) driven by the *eve* locus under the TF levels indicated in A-C. Model output is standardized to the maximum rate driven by the locus in the wildtype *trans* environment. Data for relative [mRNA] of *eve* (gray shading) is included for visual orientation within the embryo and levels are not commensurate with predicted locus output. G-H: Predicted relative [mRNA] levels (red lines) driven by the *eve* Stripe 3+7 enhancer under the TF levels indicated in A-C. Model output is standardized to the maximum rate driven by the enhancer in the wildtype *trans* environment. Data for relative [mRNA] driven by the stripe 3+7 enhancer (gray shading) is included for visual orientation within the embryo and levels are not commensurate with predicted enhancer output.

These numerical results are reminiscent of a recent experimental result showing that the locus and 3+7 enhancer respond differently to the ectopic expression of Hb driven by the *snail* promoter [29]. Under ectopic expression of Hb, stripe 7 is lost when driven by the stripe 3+7 enhancer, however when driven by the intact locus, stripe 7 is not lost and expression expands towards the anterior. Ectopic Hb leads to complex changes in the *trans* environment [60] and specific levels of transcription factors are unknown, however we are able to simulate changes in *trans* to test whether our model is consistent with these results. To this end, we set Hb levels to a spatially uniform value (Fig 6B). We find that expression driven by the 3+7 enhancer is lost (Fig 6H), but stripe 7 is not lost when driven by the entire *eve* locus (Fig 6E). We do not observe the anterior expansion of stripe 7 when only Hb expression is changed, but ectopic expression of Hb has pleiotropic effects which act to reduce levels of both Gt and Kni in the posterior of the embryo [60]. A reduction in the level of Gt (Fig 6C) in addition to ectopic Hb is sufficient to drive the anterior expansion of this stripe (Fig 6F).

### Activation by hunchback and Stat92E

It has long been recognized that *eve* is activated by broadly distributed factors [6, 7, 40]. Our model included three transcriptional activators: Bcd, Caudal (Cad), and Stat92E (Dst). Additionally, the repressor Hb is able to activate when bound near Bcd or Cad [14, 47, 61], a phenomenon called coactivation. In order to determine which factors are responsible for activation we found the percent of adaptors recruited to the TSS by each transcriptional activator (Fig 4A). We find that the majority of activation is driven by Stat92E, with a significant contribution from Hb in the anterior and posterior portions. While we do not observe large direct contribution from Bcd and Cad, these factors are responsible for the activating activity of Hb through coactivation.

### Behavior of a predicted *cis*-regulatory element

**Fig 7:**
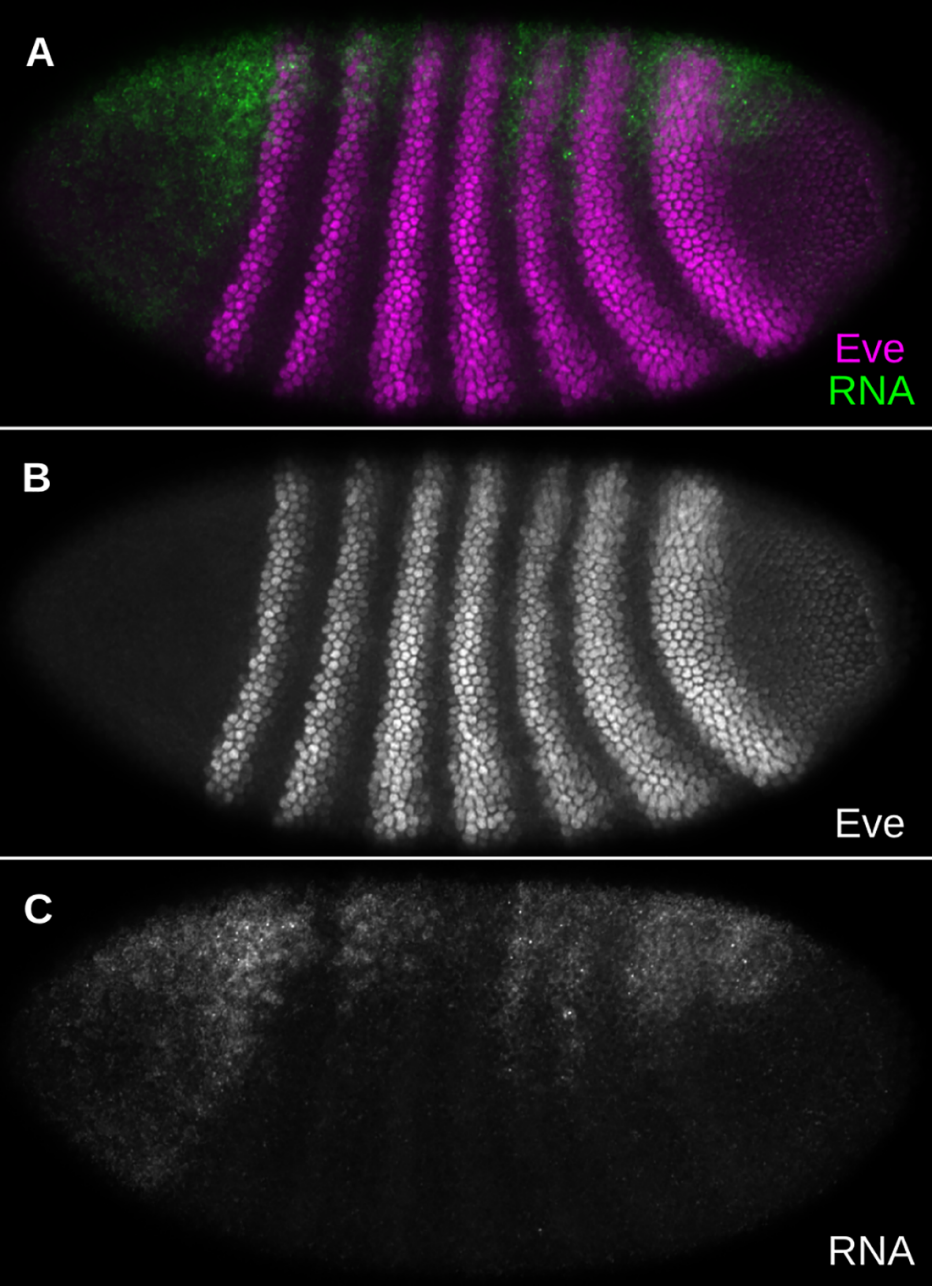
Expression driven by a predicted enhancer. A 900bp sequence, located between 3130 and 2230 bp upstream of the *eve* TSS, was placed upstream of a lacZ reporter. An embryo containing this construct at the AttP2 site [48] was stained by FISH and immunostaining with antisense lacZ probe and *α*–Eve antibody [62] respectively. The embryo was imaged in late nuclear cycle 14 with a 20x objective on a Zeiss 710 confocal microscope. A: Eve (magenta) and lacZ (green) B: Eve in grayscale C: lacZ in grayscale.

Our results indicate that most of the activation of stripe 7 is driven by a sequence upstream of the stripe 2 enhancer, between that and the stripe 3+7 enhancer elements (Fig 3). We took a 900bp fragment, located between 3130 and 2230 bp upstream of the TSS and centered on this region, and tested its ability to drive expression of *lac*Z *in vivo*. This sequence, which we call the 3130 element, drives expression dorsally overlapping stripe 2 and stripes 5 through 7 (Fig 7A and 7C). This fragment drives stronger and more ventral expression within the posterior interstripes than in the stripes themselves. Remarkably, this pattern is not observed in reporter assays for larger sequences that contain the 3130 element [4].

### Incorporation of chromatin state

**Fig 8:**
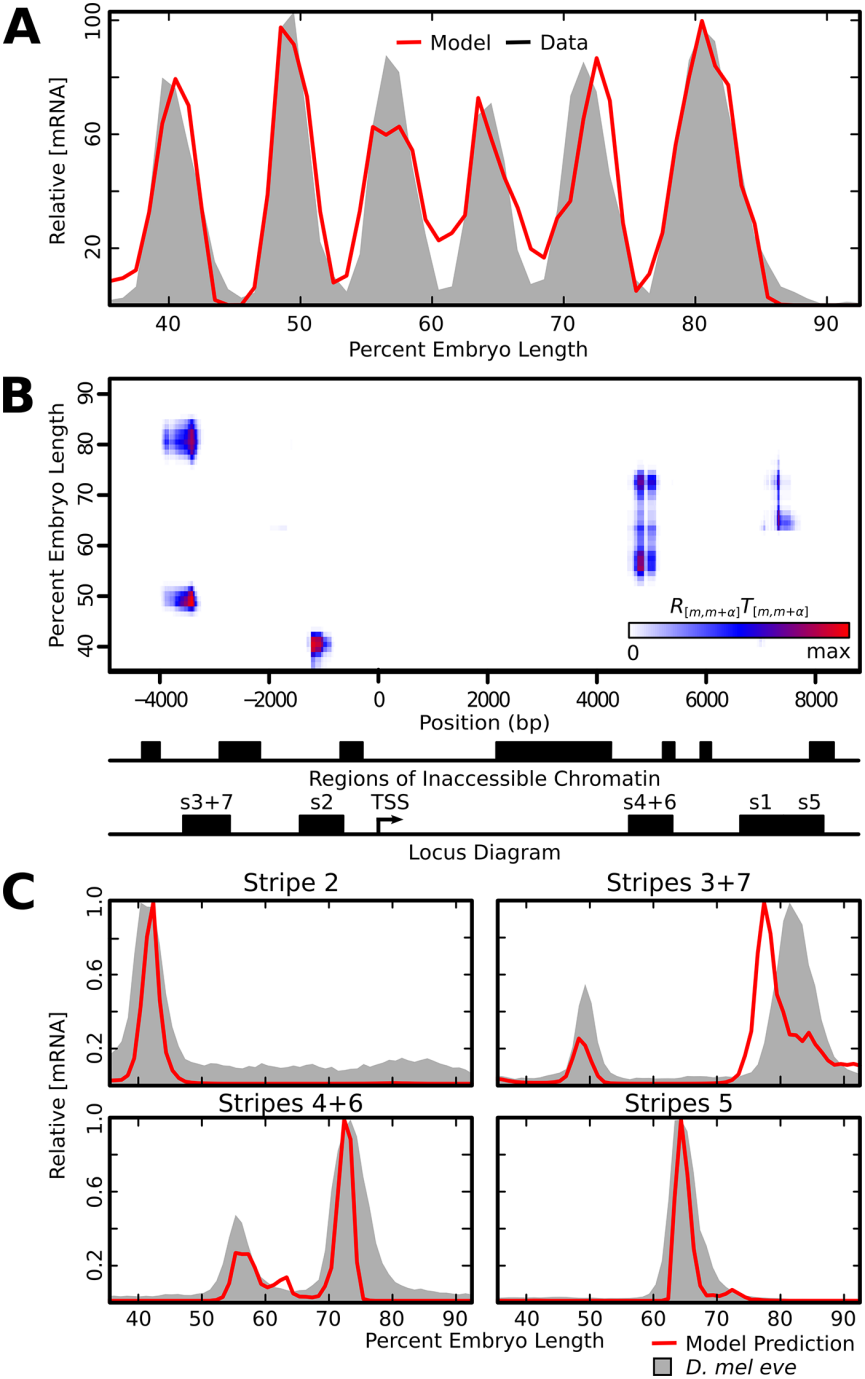
Model output after masking inaccessible chromatin. We excluded transcription factor binding within regions identified as inaccessible and retrained model parameters. A: Observed mRNA levels (gray shading) are shown together with model output (red line) after inclusion of a chromatin mask. B: Heatmap of the quantity quantity *R*_[*m*,*m*+*α*]_*T*_[*m*,*m*+*α*]_ at each nucleotide and embryo position, representing the amount each 1kb sequence, centered at that nucleotide, contributes towards total expression. The identified regions of inaccessible chromatin and locations of known enhancers are indicated on the x-axis. C: We tested the relative output of the known *eve* enhancers *in silico* using the retrained model (red lines). The relative mRNA driven by each enhancer (gray shading), is included for visual orientation within the embryo and levels are not commensurate with predicted enhancer output.

It is possible that the assay for the 3130 element is not faithful to *in vivo* expression because this fragment has been removed from its native chromatin state. Indeed, the 3130 element falls into inaccessible chromatin when assayed using either DNAse-seq [24] or FAIRE-seq [25]; moreover models of binding trained with DNAse-seq and ChIP-seq data do not predict binding in this region [63]. In order to incorporate this information, we defined accessible nucleotides to be those that are within accessible regions found using either DNAse-seq or FAIRE-seq(Fig 8B). Then we retrained the model, this time only scanning for transcription factor binding sites that were within accessible chromatin. After training, the best parameter set generated an equally good fit to data as those that did not incorporate chromatin status (Fig 8A). We no longer find expression driven by the 3130 element within the context of the intact locus (Fig 8B), but this fragment is still predicted to drive expression when removed from its native chromatin context (S4 Fig). The DNA regions that contribute to activation overlap with their corresponding enhancers (Fig 8B), and when we simulated the activity of known enhancers *in silico* each enhancer is still correctly predicted to drive expression of its corresponding stripes (Fig 8C). The identified mechanisms of stripe border control (S5 Fig) and predicted effects of ectopic Hb (S6 Fig) did not change after inclusion of chromatin accessibility data.

## Discussion

The central result of this paper is the demonstration that the enhancer structure of *eve* arises because of competition between different regions of the proximal promoter for interaction with the basal complex (Eq (2)). The competition described may reflect kinetic statistics of interactions between distally bound adaptors and the basal complex. This competition differs from steric competition for a binding site (cf Eq S4 in S1 Appendix) in that *N*_[*m*,*m*+*α*]_, unlike *q*_*i*_ (Eq S6 in S1 Appendix), depends not only on thermodynamically described interactions of TFs with the DNA but also on the protein-protein interactions which convert repressors into activators by coactivation and quench activators (Eqs S14 and S16 in S1 Appendix).

Previously, the independent action of enhancers has been explained by quenching. This short range repression mechanism allows expression to be driven by one enhancer while transcriptional repressors bind to quenched enhancers only a few hundred nucleotides away [64]. This mechanism is indeed necessary to explain the action of *eve* enhancers, but if repression occurs over short distances then low levels of bound activators over sufficiently long pieces of DNA will eventually overcome repression. Some additional mechanism must exist to prevent this domination of activation over repression. Short range repression, together with the competition of activators for interaction with the basal complex is sufficient to explain the independent action of *eve* enhancers in the context of the whole locus. Furthermore, such mechanisms may explain the nonadditive effects observed for shadow enhancers [21, 22] which are now known to be a common feature of developmentally important genes [17].

### Advancements over previous work

One previous work has modeled the regulation of *eve* by its entire regulatory sequence [16]. These authors devised a two tiered model, in which the lower tier is a previously reported enhancer model [12] which uses a thermodynamic picture of protein binding, and is capable of modeling short range repression but not coactivation. The starting parameters of this lower tier component are determined by fitting to a set of expression data from approximately 40 enhancers from 27 genes, excluding the one to be modeled. In the second tier of the model, a collection of up to 5 DNA segments (“windows”) for each expression domain is constructed as follows. Every possible DNA segment in the locus with starting points at 100 bp intervals and lengths between 500 and 2500 bp is considered. For each expression domain or stripe the 5 segments that give the best pattern for that domain are chosen. A model of the whole locus is then constructed from a weighted sum of the expression patterns driven by the segments chosen, and then the first tier parameters are retrained while keeping the DNA segments and their weights fixed. This cycle of training window weights and first tier parameters is continued until the score ceases to improve.

The chief difference between the work reported here and that reported by Samee and Sinha [16] is that those investigators started with the assumption that genes have an enhancer structure. This assumption had two consequences. First, the lower tier model of individual enhancers [12], alluded to above, is the starting point and an integral component of the model of the whole locus. The lower tier model was trained on expression data from isolated enhancers. Our previously reported models of isolated enhancers were not used to construct the whole locus model reported here, nor was expression data driven by isolated enhancers used for training.

Second, the second tier of the previously reported model assumes a one-to-one mapping between contiguous segments of DNA and expression domains, a point that is integral to the fitting procedure described above and the weighting of expression driven by DNA segments. The weights were constant over the whole embryo and only assigned to the five segments of DNA which best matched expression domains. In this work, the weighting is done not in terms of expression domains in the embryo but rather in terms of activation on the distal promoter. This is done in such a way that strongly activating distal promoter regions have stronger interactions with the basal promoter. As a consequence, the relative contributions of individual segments varies from cell to cell as the concentrations of TFs vary. This leads to competition that extends to the interstripes, and may be a reason why expression in the interstripes is higher in the previously reported work, extending to about three quarters of peak stripe expression in the interstripe between stripes 2 and 3 (see Fig 4 in [16]). Moreover, in this work the weighting by activation is always performed at single nucleotide resolution over the whole locus (Eqs (2) and (3)) rather than being limited to five segments of DNA. Another difference between the models is with respect to coactivation, which we comment on below. Although the different treatment of coactivation affects biological conclusions about *eve*, this difference arises from prior work by both groups at the enhancer level [12, 14].

### Transcriptional regulation of *eve*

A locus level understanding of gene regulation is complicated by the context dependent action of transcription factors. It has previously been shown that ectopic expression of Hb leads to the loss of *eve* stripe 7 when driven by the stripe 3+7 enhancer, but not when driven by the locus [29]. Our model includes a coactivation mechanism, where locally bound Bcd and Cad cause Hb to switch from repressor to activator [14, 47, 61]. This coactivation is required for the activation of *eve* stripe 7 within the posterior Hb domain. In the model reported here, higher spatially uniform levels of Hb expression, which presumably mimic the reported results [29], repress the stripe 3+7 enhancer by providing additional quenching from Hb sites distant from bound Cad (Fig 6H). However, the locus is still able to drive stripe 7 through the action of DNA upstream of the stripe 2 enhancer. These results indicate that coactivation by Bcd and Cad is sufficient to explain dual regulation by Hb. This mechanism was not treated by Samee *et al.* [16].

We find that Dst has a major contribution towards the activation of *eve*. Evidence in favor of this finding is afforded by the observations that stripe 3+7 expression is reduced by Dst binding site mutations [58] and that *eve* RNA levels drop by a factor of greater than 6 in embryos that lack maternal Dst [65]. Some ambiguity remains, however. Embryos lacking maternal and zygotic Dst still express seven *eve* stripes when driven from the intact *eve* locus [66], presumably at reduced levels. However, these embryos fail to drive stripe 3 from the proximal 5.2kb of the *eve* promoter. In addition to highlighting another difference between fragments driving reporter expression and the intact locus, these results indicate the likely presence of other widespread activators. Possible candidates include Zelda [67], Trithorax-like [68] and Dicheate [69, 70]. Of these, Zelda has reported to act through modification of chromatin state [71, 72], which we have treated directly. In this work we did not include these additional wide spread activators because their functional roles cannot be distinguished without further experimental information. Such experimental information might take the form of quantitative assays of *eve* expression in embryos lacking maternal and zygotic contributions of each of these factors in various combinations. Alternatively, a defined synthetic promoter could be built up by systematically adding binding sites for one such factor at a time.

We find that enhancers do not necessarily follow the same regulatory logic as the intact locus. When a sub-sequence of the intact locus is placed into an enhancer-reporter assay it is removed from the context of the locus. The enhancer may not contain all the sites or regulatory interactions present in extended sequence and thus will not follow the same logic as the locus. In this work we identify a specific case with regards to the regulation of stripe 2. When driven by MSE2, the posterior border of stripe 2 is more strongly regulated by Bcd and Hb than when stripe 2 is driven by the intact locus, which explains the lack of posterior expansion when Kr binding is disrupted [6]. This is consistent with previous reports on other enhancers. For instance, in Kni– embryos the posterior of stripe 3 and the anterior border of stripe 6 are abolished when expression is driven by MSE3, while these borders remain present when driven by the intact locus [7, 55]. Additionally, for features whose regulation is distributed, as in stripe 7, each CRM uses a separate set of factors to generate function. As such, changes to the environment in *trans* will have different effects than observed on either element alone.

### Consequences of enhancer competition

Competition for the basal complex has direct consequences for *eve* expression. The expression patterns driven by individual enhancers are broader than than the same stripes driven by the intact locus [29]. These broad expression domains driven by individual enhancers overlap at the interstripes. If expression is additive at these positions, there will be poor repression in *eve* interstripes. However, competing enhancers will drive expression at levels less than the sum of the rates driven by either enhancer alone. Thus, enhancer competition is sufficient to explain how sharp stripes are driven by broadly expressed enhancers.

Studies of how transcription rate varies with respect to the positioning and separation between bound activators will be required to distinguish between different modes of enhancer competition, additivity, or cooperativity. Specifically, the model proposed in this work suggests that if two activators are bound adjacently, these activators will synergistically activate transcription through cooperative action on the basal complex. If these activators are then separated by increasing lengths of neutral DNA we expect that transcription rate will decline linearly up to a distance of *α* (Eqs (1)–(3)), at which point there will be steric hindrance preventing simultaneous interaction with the basal complex. If the rate does not decline, it implies that sequences do not compete, and that instead intervening DNA can be looped out. Such experiments could be performed by varying the relative positions and orientations of shadow enhancers acting on a common promoter.

### Latent enhancers

We identify a case in which a DNA fragment had regulatory activity in a reporter assay, but not in the intact locus. Our model predicted the existence of a new regulatory element in the *eve* locus, however when we tested the activity of this fragment *in vivo* we found that it drove expression in an unexpected pattern that is not a subset of the expression pattern driven by the *eve* locus. For that reason we conclude that this fragment is not active in the intact *eve* locus and that placing it upstream of a reporter has revealed latent function. We hypothesized that this fragment may lie within inaccessible chromatin. Indeed, when we only model binding sites within accessible chromatin, we no longer predict expression driven by this fragment in the intact locus. This result highlights the importance of studying intact loci in addition to isolated enhancers and indicates that incorporation of chromatin accessibility increases the accuracy and utility of regulatory models.

### Calculation of fractional occupancy

Transcription factor occupancy is critical to the control of gene regulation, but this quantity can be expensive to compute. As the length of DNA and the number of interacting bound transcription factors increases, the number of configurations and hence the computation time increases exponentially. Other groups have adopted dynamic programming approaches where the computation time increases linearly with the number of sites, but they do not calculate the occupancy of individual sites [12] or use some type of Gibbs sampler [10, 36, 63]. The algorithm presented here computes pairwise cooperativity and scales linearly with the number of binding sites. This algorithm has similar performance to one previously reported [73], but that algorithm is incompatible with the pairwise cooperativity observed with Bcd. Our algorithm allows thermodynamic models to be applied to genomic scale data with low thresholds for transcription factor binding, and we believe it to be a potentially useful technical development.

### Generalizability of this approach

Our analysis of *eve* depended on having the DNA sequence and chromatin accessibility of the locus, expression data from the locus over a range of cell types comparable to what is seen *in vivo*, a complete set of regulators (although as we discussed above, there is some ambiguity as to the full set of activators), a set of PWMs for these regulators, an understanding of the extent of the locus, and knowledge of the functional roles of the TFs used in the models. Overall, most or all of this information is already available in numerous systems or can be obtained, at least in principle, by high-throughput techniques. Entire genome sequences are now available for a large number of organisms together with functional data including chromatin accessibility [74, 75] across numerous tissues and cell lines. Expression data could be achieved through RNA-seq on a carefully curated set of cell lines, or alternatively from single-cell techniques on more homogeneous tissues. The cells from which expression data is obtained must also be subjected to transcriptome or proteome analysis to reveal the TFs present. The extent of the locus can be obtained my mapping insulator elements [76] or using chromosome conformation assays [77]. Curated sets of PWMs for TFs are readily available [78], and TF roles and interactions can be inferred from data [79] or learned by comparing the model results of all possible perturbations of functional roles [80]. While this list appears imposing, all of the assays mentioned are regularly performed and the challenge is to integrate them together in an effective high-throughput approach. The study reported here provides a proof of concept for such future investigations.

## Supporting information

S1 Fig Model fits without enhancer competition. A: The transcription model, given by Eqs S1-S17 (in S1 Appendix) and Eq (3), was trained to the expression pattern of *even-skipped*. Percent embryo length(*x*-axis) is measured from the anterior pole. The identity of each *eve* stripe is indicated. The model (red line) is able to achieve good fits to data (black line). B-E: Using the model shown in A, we predicted the [mRNA] driven by four enhancers that have previously been shown to drive each of the stripes (red lines). The identity of each sequence is labeled. Sequence coordinates for each enhancer are reported in Materials and Methods. The locus data that corresponds to each stripe is shown with black lines. F-I: We trained the model to the four *eve* enhancers driving their respective portion of the locus pattern. This model output (red lines) achieves good fits to data (black lines). J: We used the model shown in F-I to predict expression driven by the entire *eve* locus. Predicted output (red line); Data (black line).
(TIF)

S2 Fig Rate driven by stripe 2 enhancers MSE2 and S2E. The 480 bp MSE2 fragment and the 800bp S2E were placed upstream of lacZ and cloned into the AttP2 site in *Drosophila*. Mean fluorescent in-situ hybridization (FISH) intensity at nuclear cycle 14 timepoint 6 is reported with S2E in solid lines and MSE2 in dashed lines. 15 embryos containing S2E were imaged, giving between 47 and 63 nuclei per AP position. 8 embryos containing MSE2 were imaged, giving between 26 and 37 nuclei per AP position. Peak expression of S2E is 5.5 times greater than that of MSE2, despite only containing 320 additional bases.
(TIF)

S3 Fig Best model fit using 500bp window. A: the model output (red line) and data (gray shading) for the best fit to data. B: Heatmap of the quantity quantity *R*_[*m*,*m*+*α*]_*T*_[*m*,*m*+*α*]_ at each nucleotide and embryo position, representing the amount each 1kb sequence, centered at that nucleotide, contributes towards total expression. The locations of known enhancers are indicated on the *x*-axis. C: We tested the relative output of the known *eve* enhancers *in silico* using the retrained model (red lines). The relative mRNA driven by individual enhancers (gray shading), is included for visual orientation within the embryo and levels are not commensurate with predicted enhancer output.
(TIF)

S4 Fig Prediction of e3130 element in model with accessibility. The model was trained as described in Fig 1, except binding sites were only called within regions of accessible chromatin. We predicted the activity of the 3130 element *in silico* to test its activity outside of its native chromatin context. The relative model output (red line) is plotted with *eve* mRNA. The relative mRNA driven by the locus (gray shading) is included for visual orientation within the embryo and levels are not commensurate with predicted enhancer output.
(TIF)

S5 Fig Mechanisms of repression in locus model with accessibility. The model was trained as described in Fig 1, except binding sites were only called within regions of accessible chromatin. Cumulative line graph showing the change in [mRNA] caused by a change in concentration of each TF (y-axis) at each embryo position (*x*-axis).
(TIF)

S6 Fig Predicted effects of ectopic Hb in model with accessibility. The model was trained as described in Fig 1, except binding sites were only called within regions of accessible chromatin. A: The measured relative levels of Hb and Gt (y-axis) from 35.5% to 92.5% embryo length (*x*-axis). B: Simulated relative levels of Hb and Gt. Hb is set to a spatially uniform value and Gt is unchanged from A. C: Simulated relative levels of Hb and Gt. Hb is set to a spatially uniform value and Gt is reduced by 40%. D-F: Predicted relative [mRNA] levels (red lines) driven by the *eve* locus under the TF levels indicated in A-C. Model output is standardized to the maximum rate driven by the locus in the wildtype *trans* environment. Data for relative [mRNA] of *eve* (gray shading) is included for visual orientation within the embryo and levels are not commensurate with predicted locus output. G-H: Predicted relative [mRNA] levels (red lines) driven by the *eve* Stripe 3+7 enhancer under the TF levels indicated in A-C. Model output is standardized to the maximum rate driven by the enhancer in the wildtype *trans* environment. Data for relative [mRNA] driven by the stripe 3+7 enhancer (gray shading) is included for visual orientation within the embryo and levels are not commensurate with predicted enhancer output.
(TIF)

S7 FigNumerical partial derivative estimates.The partial derivative $\frac{\partial R}{[\text{TF]}}$ was estimated for each modeled TF using the symmetric difference quotient $\frac{f(x+h)-f(x-h)}{2h}$, at each position in the embryo, where *h* is the change in fluorescence of the TF in question over adjacent nuclei. Estimates are robust over values of *h* from 10^−1^ through 10^−11^.(TIF)

S8 Fig Best three model fits after repeating the optimization procedure. We repeated the optimization procedure an additional 80 times. The best three model fits have similar predictions to the model used to generate figures in the main text. We report predictions for the three parameter sets with the lowest score. A-C: the model output (red line) and data (gray shading) for the top three parameter sets respectively. D-F: Heatmap of the quantity quantity *R*_[*m*,*m*+*α*]_*T*_[*m*,*m*+*α*]_ at each nucleotide and embryo position, representing the amount each 1kb sequence, centered at that nucleotide, contributes towards total expression. The locations of known enhancers are indicated on the *x*-axis. G-I: We tested the relative output of the known *eve* enhancers *in silico* using the retrained model (red lines). The relative mRNA driven by individual enhancers (gray shading), is included for visual orientation within the embryo and levels are not commensurate with predicted enhancer output.
(TIF)

S9 Fig Best three model fits after repeating the optimization procedure. We repeated the optimization procedure an additional 80 times for fits incorporating chromatin data. The best three model fits have similar predictions to the model used to generate figures in the main text. We report predictions for the three parameter sets with the lowest score. A-C: the model output (red line) and data (gray shading) for the top three parameter sets respectively. D-F: Heatmap of the quantity quantity *R*_[*m*,*m*+*α*]_*T*_[*m*,*m*+*α*]_ at each nucleotide and embryo position, representing the amount each 1kb sequence, centered at that nucleotide, contributes towards total expression. The identified regions of inaccessible chromatin and locations of known enhancers are indicated on the *x*-axis. G-I: We tested the relative output of the known *eve* enhancers *in silico* using the retrained model (red lines). The relative mRNA driven by individual enhancers (gray shading), is included for visual orientation within the embryo and levels are not commensurate with predicted enhancer output.
(TIF)

S1 File Florescence levels and standard deviations for *even-skipped* mRNA at T6. (XLS)

S2 File Excel file contain all parameter sets and model scores, as well as parameter search space. (XLS)

S1 Appendix Supplementary methods. (PDF)
