# Correction: A sequence level model of an intact locus predicts the location and function of nonadditive enhancers

**DOI:** 10.1371/journal.pone.0197211

**Published:** 2018-05-07

**Authors:** Kenneth A. Barr, John Reinitz

There is an error in the fifth sentence under the sub-heading “Sequence selection” in the Materials and methods section. The correct sentence is: For this figure, the stripe 2 enhancer, sometimes called S2E, is the 700bp sequence spanning conserved blocks A and B first reported in [37] and has dm3 coordinates 2R:5865217–5866014.

There is an error in the fifth sentence under the sub-heading “An enhancer competition model” in the Results section. The correct sentence is: Indeed, we find that when the 480 bp minimal stripe 2 element of eve (MSE2) is extended by 218 bp there is a five fold increase in transcription rate (S2 Fig).

The caption of S2 Fig must be modified to reflect this change. Please see the corrected version here.

## Supporting information

S2 Fig Rate driven by stripe 2 enhancers MSE2 and S2E. The 480 bp MSE2 fragment and the 698 bp S2E (dm3 coordinates 2R:5865217–5865913) were placed upstream of lacZ and cloned into the AttP2 site in *Drosophila*. Mean fluorescent in-situ hybridization (FISH) intensity at nuclear cycle 14 timepoint 6 is reported with S2E in solid lines and MSE2 in dashed lines. 15 embryos containing S2E were imaged, giving between 47 and 63 nuclei per AP position. 8 embryos containing MSE2 were imaged, giving between 26 and 37 nuclei per AP position. Peak expression of S2E is 5.5 times greater than that of MSE2, despite only containing 218 additional bases.
(TIF)
