# Supplementary figures and images for: A sequence level model of an intact locus predicts the location and function of nonadditive enhancers
Source: PLoS One. 2017 Jul 17;12(7):e0180861. doi: 10.1371/journal.pone.0180861 (PMC5513433; doi:10.1371/journal.pone.0180861)

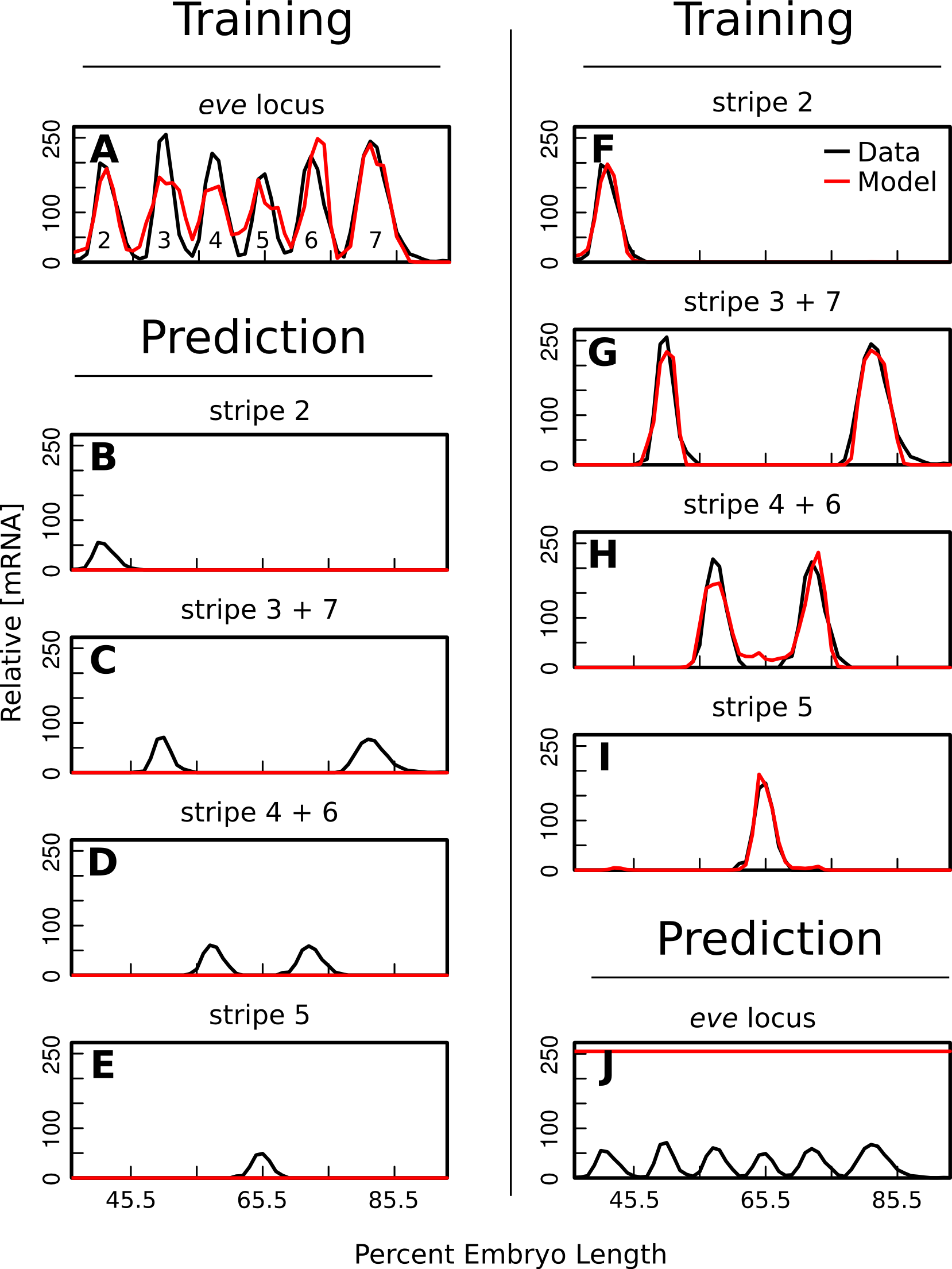

Supplement: S1 Fig — A: The transcription model, given by Eqs S1-S17 (in S1 Appendix) and Eq (3), was trained to the expression pattern of even-skipped. Percent embryo length(x-axis) is measured from the anterior pole. The identity of each eve stripe is indicated. The model (red line) is able to achieve good fits to data (black line). B-E: Using the model shown in A, we predicted the [mRNA] driven by four enhancers that have previously been shown to drive each of the stripes (red lines). The identity of each sequence is labeled. Sequence coordinates for each enhancer are reported in Materials and Methods. The locus data that corresponds to each stripe is shown with black lines. F-I: We trained the model to the four eve enhancers driving their respective portion of the locus pattern. This model output (red lines) achieves good fits to data (black lines). J: We used the model shown in F-I to predict expression driven by the entire eve locus. Predicted output (red line); Data (black line). (TIF) [file pone.0180861.s001.tif]

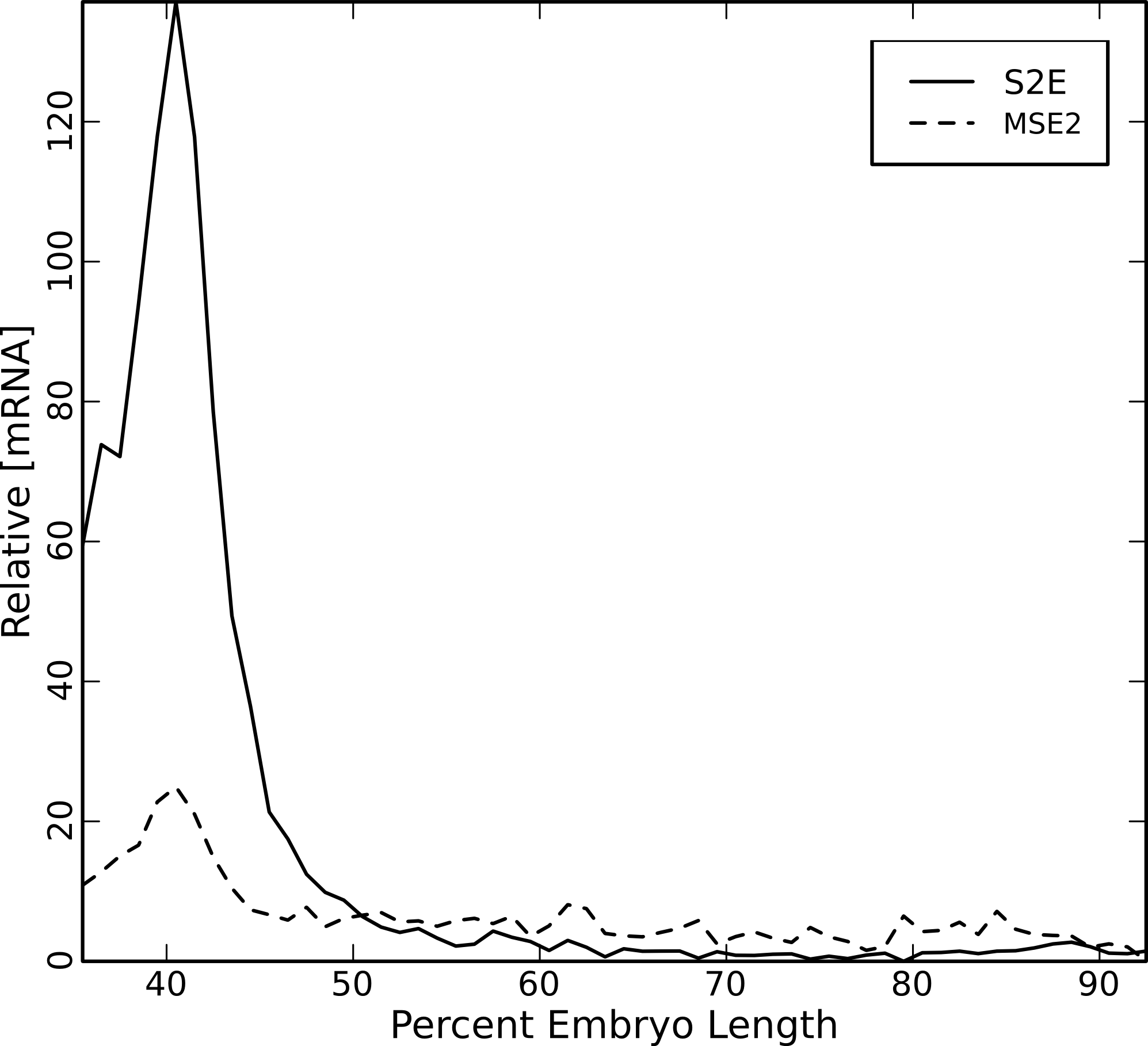

Supplement: S2 Fig — The 480 bp MSE2 fragment and the 800bp S2E were placed upstream of lacZ and cloned into the AttP2 site in Drosophila. Mean fluorescent in-situ hybridization (FISH) intensity at nuclear cycle 14 timepoint 6 is reported with S2E in solid lines and MSE2 in dashed lines. 15 embryos containing S2E were imaged, giving between 47 and 63 nuclei per AP position. 8 embryos containing MSE2 were imaged, giving between 26 and 37 nuclei per AP position. Peak expression of S2E is 5.5 times greater than that of MSE2, despite only containing 320 additional bases. (TIF) [file pone.0180861.s002.tif]

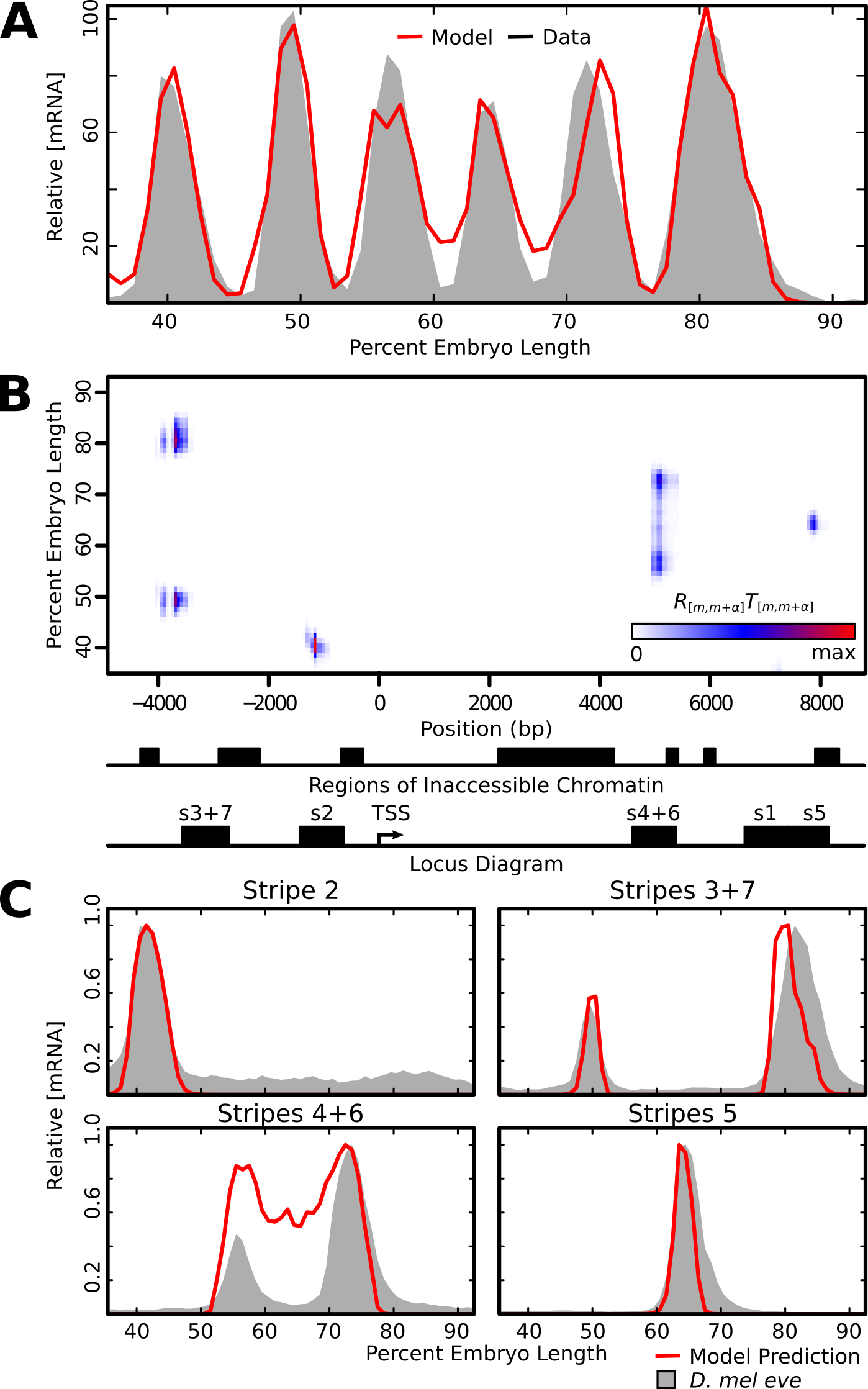

Supplement: S3 Fig — A: the model output (red line) and data (gray shading) for the best fit to data. B: Heatmap of the quantity quantity R[m,m+α]T[m,m+α] at each nucleotide and embryo position, representing the amount each 1kb sequence, centered at that nucleotide, contributes towards total expression. The locations of known enhancers are indicated on the x-axis. C: We tested the relative output of the known eve enhancers in silico using the retrained model (red lines). The relative mRNA driven by individual enhancers (gray shading), is included for visual orientation within the embryo and levels are not commensurate with predicted enhancer output. (TIF) [file pone.0180861.s003.tif]

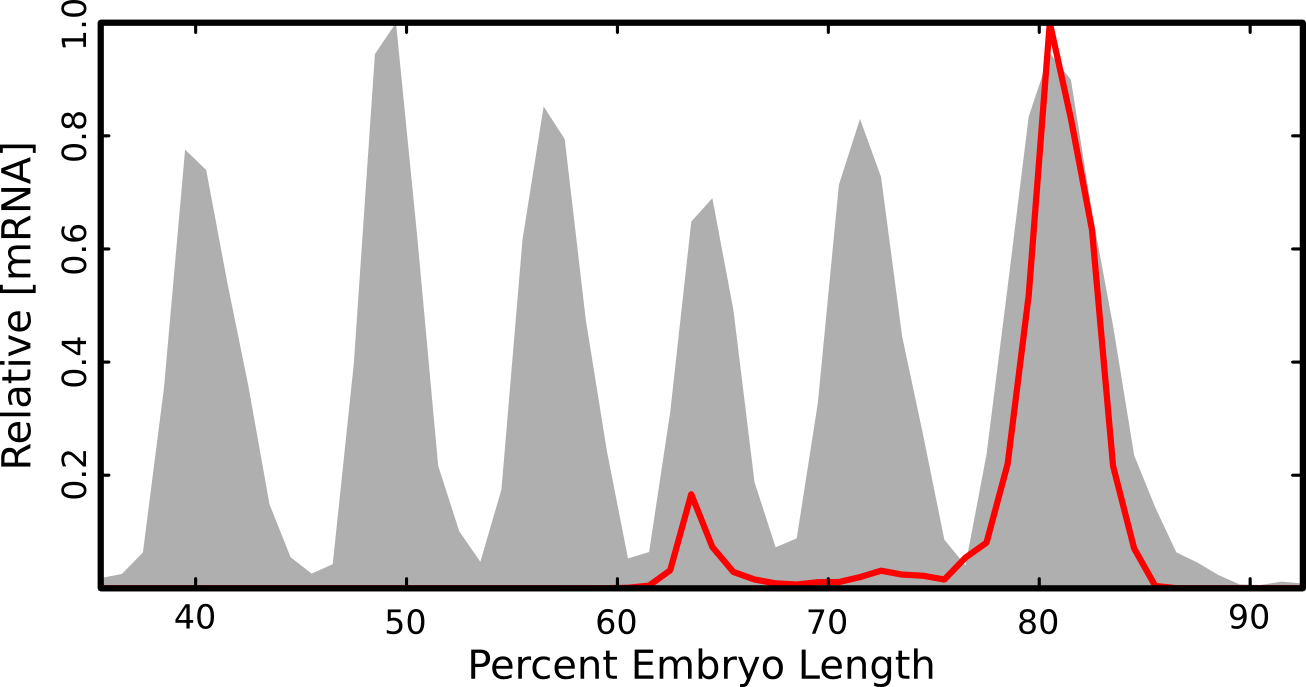

Supplement: S4 Fig — The model was trained as described in Fig 1, except binding sites were only called within regions of accessible chromatin. We predicted the activity of the 3130 element in silico to test its activity outside of its native chromatin context. The relative model output (red line) is plotted with eve mRNA. The relative mRNA driven by the locus (gray shading) is included for visual orientation within the embryo and levels are not commensurate with predicted enhancer output. (TIF) [file pone.0180861.s004.tif]

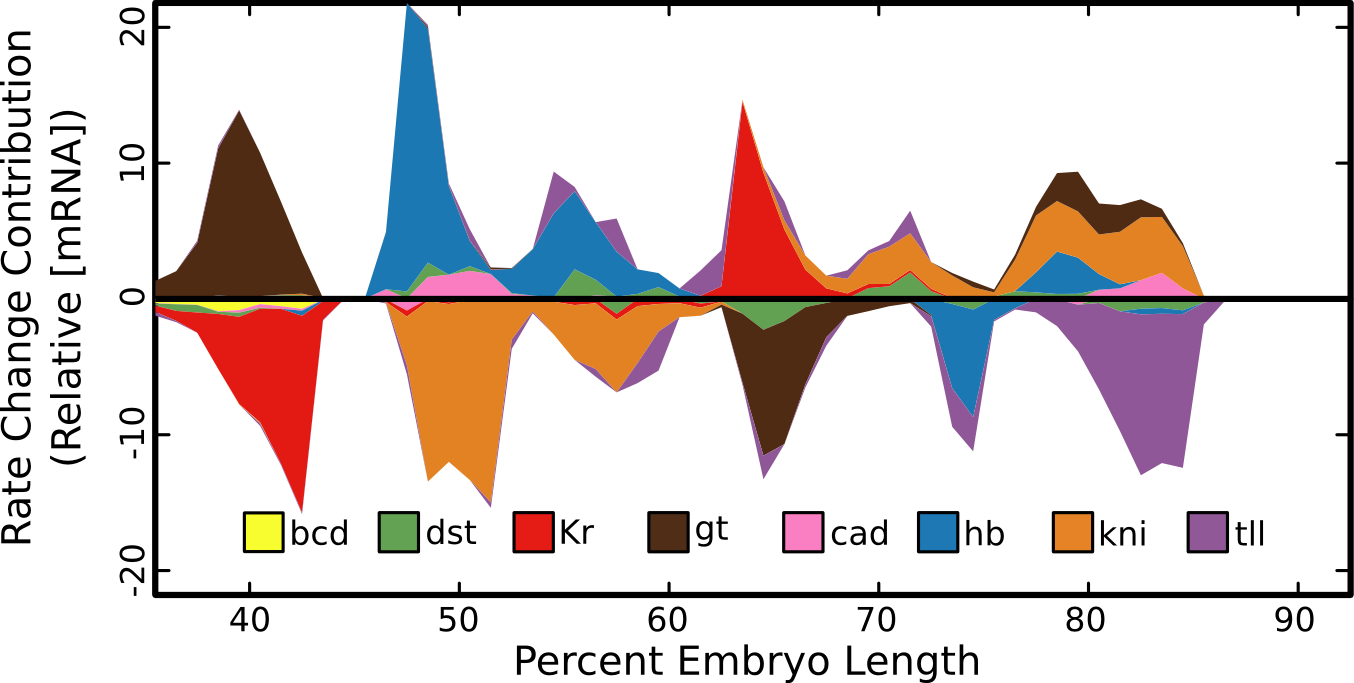

Supplement: S5 Fig — The model was trained as described in Fig 1, except binding sites were only called within regions of accessible chromatin. Cumulative line graph showing the change in [mRNA] caused by a change in concentration of each TF (y-axis) at each embryo position (x-axis). (TIF) [file pone.0180861.s005.tif]

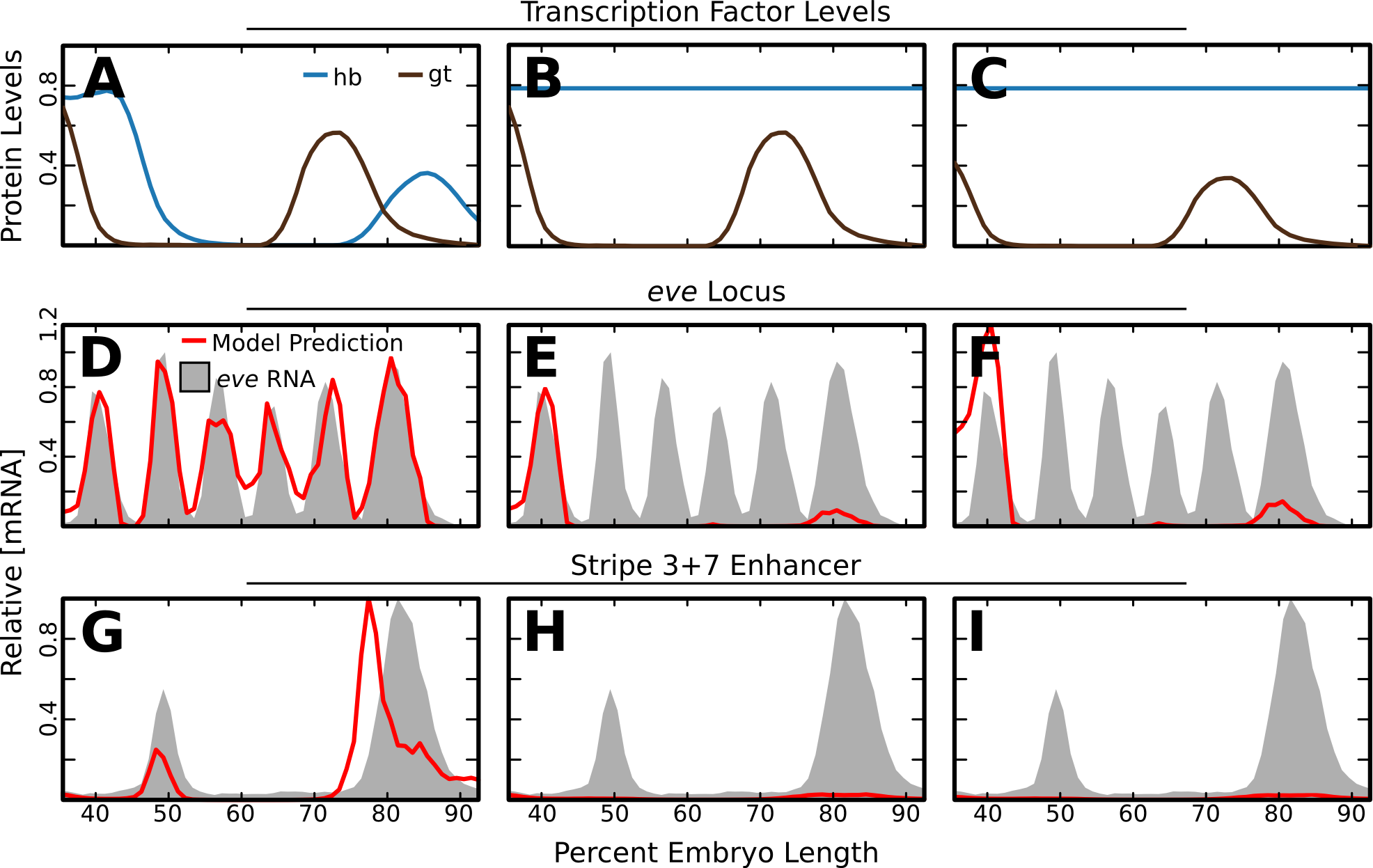

Supplement: S6 Fig — The model was trained as described in Fig 1, except binding sites were only called within regions of accessible chromatin. A: The measured relative levels of Hb and Gt (y-axis) from 35.5% to 92.5% embryo length (x-axis). B: Simulated relative levels of Hb and Gt. Hb is set to a spatially uniform value and Gt is unchanged from A. C: Simulated relative levels of Hb and Gt. Hb is set to a spatially uniform value and Gt is reduced by 40%. D-F: Predicted relative [mRNA] levels (red lines) driven by the eve locus under the TF levels indicated in A-C. Model output is standardized to the maximum rate driven by the locus in the wildtype trans environment. Data for relative [mRNA] of eve (gray shading) is included for visual orientation within the embryo and levels are not commensurate with predicted locus output. G-H: Predicted relative [mRNA] levels (red lines) driven by the eve Stripe 3+7 enhancer under the TF levels indicated in A-C. Model output is standardized to the maximum rate driven by the enhancer in the wildtype trans environment. Data for relative [mRNA] driven by the stripe 3+7 enhancer (gray shading) is included for visual orientation within the embryo and levels are not commensurate with predicted enhancer output. (TIF) [file pone.0180861.s006.tif]

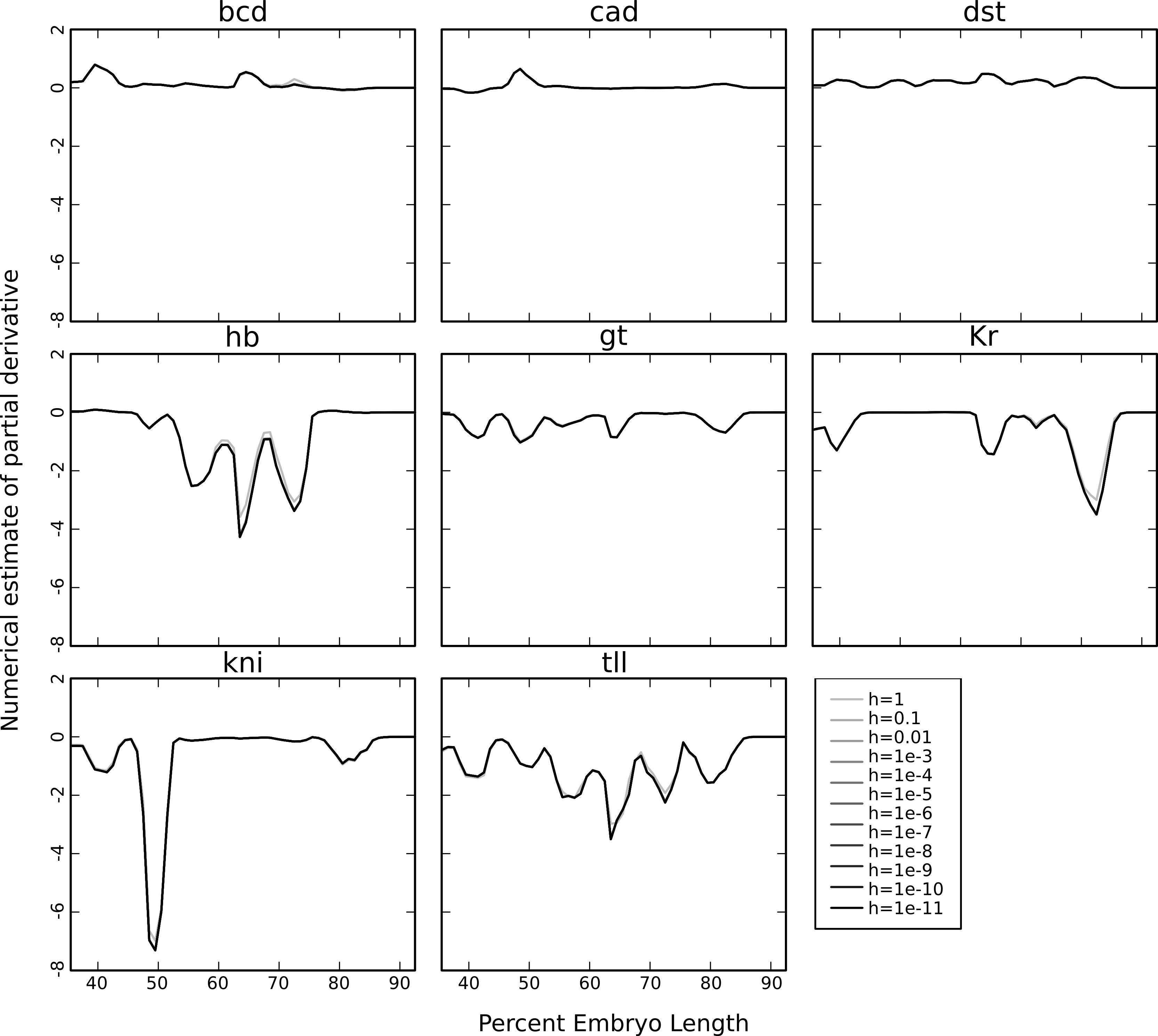

Supplement: S7 Fig — The partial derivative ∂R[TF] was estimated for each modeled TF using the symmetric difference quotient f(x+h)-f(x-h)2h, at each position in the embryo, where h is the change in fluorescence of the TF in question over adjacent nuclei. Estimates are robust over values of h from 10−1 through 10−11. (TIF) [file pone.0180861.s007.tif]

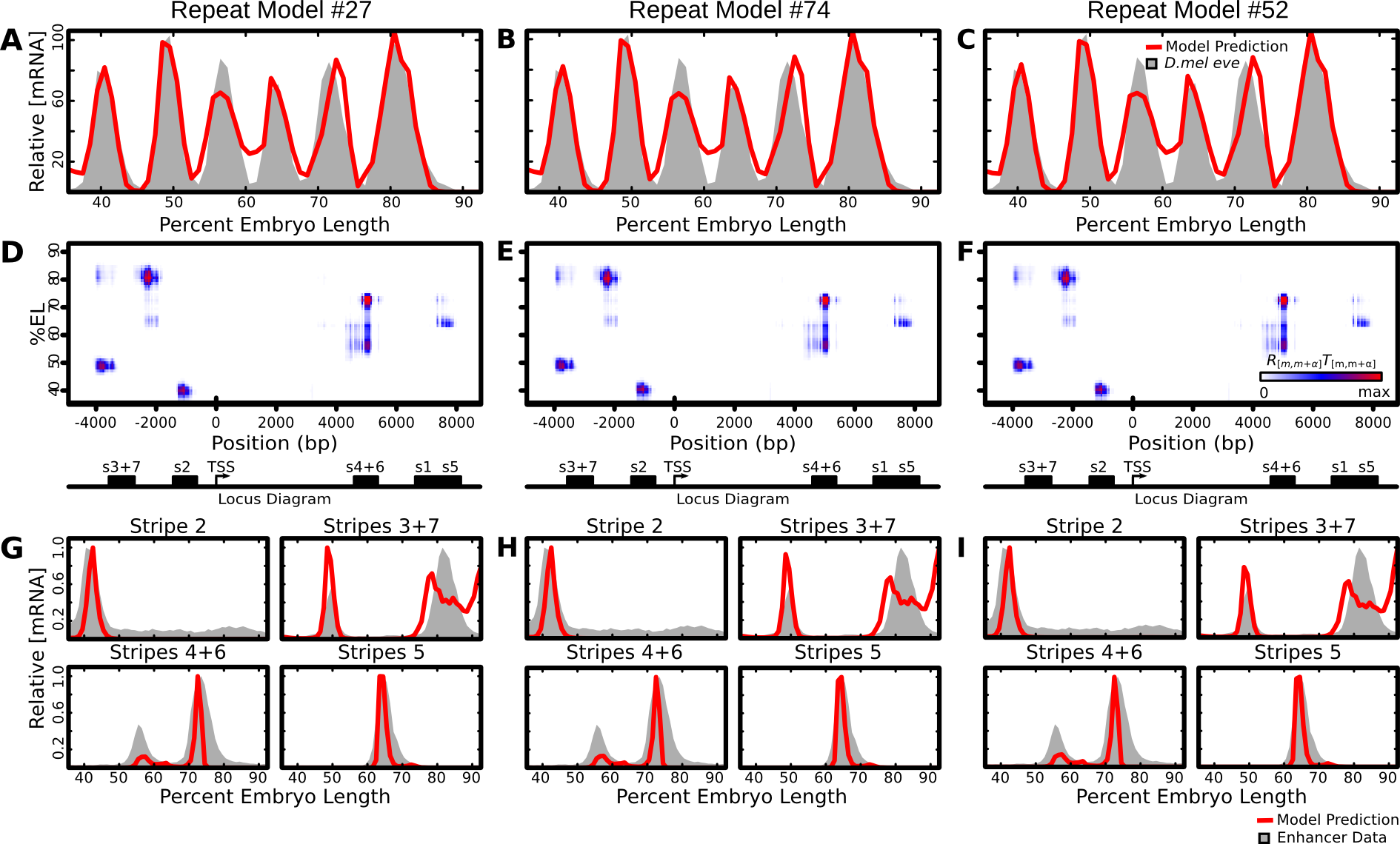

Supplement: S8 Fig — We repeated the optimization procedure an additional 80 times. The best three model fits have similar predictions to the model used to generate figures in the main text. We report predictions for the three parameter sets with the lowest score. A-C: the model output (red line) and data (gray shading) for the top three parameter sets respectively. D-F: Heatmap of the quantity quantity R[m,m+α]T[m,m+α] at each nucleotide and embryo position, representing the amount each 1kb sequence, centered at that nucleotide, contributes towards total expression. The locations of known enhancers are indicated on the x-axis. G-I: We tested the relative output of the known eve enhancers in silico using the retrained model (red lines). The relative mRNA driven by individual enhancers (gray shading), is included for visual orientation within the embryo and levels are not commensurate with predicted enhancer output. (TIF) [file pone.0180861.s008.tif]

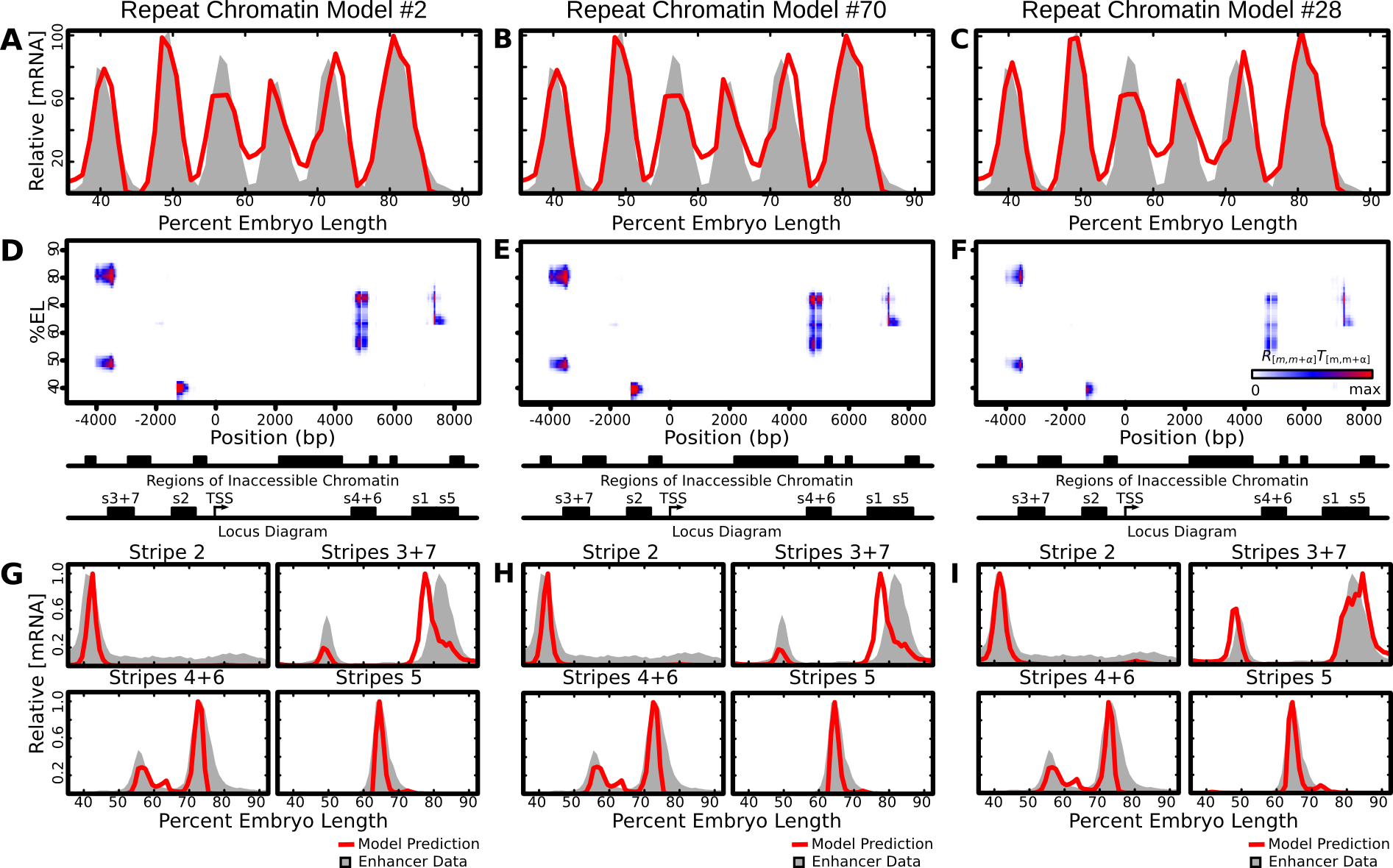

Supplement: S9 Fig — We repeated the optimization procedure an additional 80 times for fits incorporating chromatin data. The best three model fits have similar predictions to the model used to generate figures in the main text. We report predictions for the three parameter sets with the lowest score. A-C: the model output (red line) and data (gray shading) for the top three parameter sets respectively. D-F: Heatmap of the quantity quantity R[m,m+α]T[m,m+α] at each nucleotide and embryo position, representing the amount each 1kb sequence, centered at that nucleotide, contributes towards total expression. The identified regions of inaccessible chromatin and locations of known enhancers are indicated on the x-axis. G-I: We tested the relative output of the known eve enhancers in silico using the retrained model (red lines). The relative mRNA driven by individual enhancers (gray shading), is included for visual orientation within the embryo and levels are not commensurate with predicted enhancer output. (TIF) [file pone.0180861.s009.tif]
