# Supplementary material for: A sequence level model of an intact locus predicts the location and function of nonadditive enhancers
Source: PLoS One. 2017 Jul 17;12(7):e0180861. doi: 10.1371/journal.pone.0180861 (PMC5513433; doi:10.1371/journal.pone.0180861)
Supplement: S1 Appendix — (PDF) [file pone.0180861.s012.pdf]

Supplementary Methods

Gene Regulation Model

The effective number of bound transcriptional adaptors on DNA is calculated in 5 steps: (1) calculation of binding affinity across DNA sequence and designation of binding sites, (2) calculation of fractional occupancy of factors at each binding site, (3) coactivation of Hunchback by locally bound Bicoid or Caudal, (4) quenching of activators by locally bound repressors, (5) weighted summation of unquenched activators over the length of DNA sequence. Finally, the rate of transcription induced by a DNA fragment is related to the number of bound coactivators by a diffusion limited Arrhenius rate law.

Binding Affinity Position weight matrixes (PWMs) describe the relative sequence preferences of DNA-binding proteins. Given a PWM, the log-likelihood that an observed sequence i—spanning bases m though n—is a binding site for transcription factor a (which we indicate with index $i[m,n;a]$) is given by

$$S_{i[m,n;a]} = \sum_{k=m}^{n} \ln\left(\frac{P_a(k-m, j_k)}{P_{\mathrm{bg}}(j_k)}\right) \tag{S1}$$

where j_k is the nucleotide observed at position k, $P_a(k-m, j)$ is the probability of observing this nucleotide at position $k-m$ in a binding site for factor a, and $P_{\mathrm{bg}}(j_k)$ is the probability of observing this nucleotide in the null distribution. For the background distribution we use the frequencies of nucleotides in the *Drosophila* genome ($P_{\mathrm{bg}}(A) = P_{\mathrm{bg}}(T) = 0.297$, $P_{\mathrm{bg}}(C) = P_{\mathrm{bg}}(G) = 0.203$). For this work we only consider binding sites that have a score greater than zero, representing the point where sequences are equally likely to come from real binding sites as from the background distribution.

This score can be used to calculate the sequence-specific binding affinity $K_{i[m,n;a]}$, which is given by

$$K_{i[m,n;a]} = K_a^{\max} \exp\left(\frac{S_i - S_a^{\max}}{\lambda_a}\right) \tag{S2}$$

where $S_a^{\max}$ is the maximum score possible for the PWM of factor a and λ_a is a proportionality constant[1]. While we do not know absolute concentrations of transcription factors, fluorescence scales linearly with concentration [2]. We need a parameter that scales fluorescence for factor a to true concentration v_a. We call this parameter $v_a^{\max}$. Wherever this parameter occurs it is always multiplied by a K, so we cannot separate the parameter $v_a^{\max}$ from $K_a^{\max}$. In light of this, we introduce a compound parameter, A_a, that scales the product of relative affinities and fluorescence measurements to true affinities and concentrations.

$$A_a = v_a^{\max} K_a^{\max} \tag{S3}$$

Sites may be bound specifically, or non-specifically. The occupancy of a specifically bound site at fluorescence v_a^{fl} is given by

$$f_{i[m,n;a]}^{\text{sp}} = \frac{K_{i[m,n;a]} v_a^{\text{fl}} A_a}{1 + K_a^{\text{ns}} v_a^{\text{fl}} A_a + K_{i[m,n;a]} v_a^{\text{fl}} A_a} \tag{S4}$$

where K_a^{ns} is the relative nonspecific affinity of factor a. We calculate an effective binding affinity $K_{i[m,n;a]}^{\text{ef}}$ that incorporates this non-specific binding affinity by solving the relation

$$\frac{K_{i[m,n;a]}^{\text{ef}} v_a^{\text{fl}} A_a}{1 + K_{i[m,n;a]}^{\text{ef}} v_a^{\text{fl}} A_a} = \frac{K_{i[m,n;a]} v_a^{\text{fl}} A_a}{1 + K_a^{\text{ns}} v_a^{\text{fl}} A_a + K_{i[m,n;a]} v_a^{\text{fl}} A_a}$$

and find

$$K_{i[m,n;a]}^{\text{ef}} = \frac{K_{i[m,n;a]}}{K_a^{\text{ns}} v_a^{\text{fl}} A_a + 1} \tag{S5}$$

This non-specific binding affinity has been shown to be approximately three orders of magnitude smaller than the maximum specific binding energy for eukaryotic transcription factors[3], and because the calculated affinities are relative affinities with a maximum of one, we fix this non-specific affinity to 0.001.

Fractional Occupancy The fractional occupancy of a binding site is given by the ratio of the Boltzmann weights of all binding states that contain a bound transcription factor, divided by the weights all binding states, known as the partition function. The number of states grows exponentially with the number of binding sites and computing directly on all possible binding states can quickly become impossible. To solve this we use dynamic programming. First, the binding sites, indexed by $i[m_i, n_i; a_i]$, are sorted such that i increases monotonically with n in the 5' to 3' direction. For simplicity, we define the quantity

$$q_i = K^{\text{ef}}_{i[m,n;a]} v^{\text{fl}}_a A_a \tag{S6}$$

Additionally, we define a function that specifies the index of the last non-competing binding site

$$h\big(i[m,n;a]\big) = \max_{n_k \leq m_i} \big(k[m,n;a]\big) \tag{S7}$$

We find a recurrence relation where the weight Z_i of all states through the site i is a function of the weight of all states through site $i-1$, with the boundary condition $Z_0 = 1$

$$Z_i = Z_{i-1} + q_i Z_{h(i)} + \sum_{k=1}^{i-1} q_i q_k w(i,k) Z_{h(k)}, \tag{S8}$$

where $w(i,k)$ is the cooperative interaction strength between sites i and k. We store the partial partition function

$$Z^{\text{nc}}_i = q_i Z_{h(i)}, \tag{S9}$$

which holds the weight of all currently observed states bound by i, in which i is not bound cooperatively, and

$$Z^{\text{c}}_i = \sum_{k=1}^{i-1} q_i q_k w(i,k) Z_{h(k)}, \tag{S10}$$

which holds the weight of all currently observed states bound by i, in which i is bound cooperatively.

We calculate the array of weight sums Z_i in both the forward (5' to 3') and reverse (3'

to 5') direction across DNA and denote this Z_i^-, and Z_i^+. Additionally, we store partial partition functions in each direction $Z_i^{\mathrm{nc}+}$, $Z_i^{\mathrm{c}+}$, $Z_i^{\mathrm{nc}-}$, and $Z_i^{\mathrm{c}-}$. To recover the fractional occupancy of site i, given by the weight of all bound states, divided by all states, we calculate

$$f_{i[m,n;a]} = \frac{Z_i^{\mathrm{nc}+} Z_i^{\mathrm{c}-} + Z_i^{\mathrm{c}+} Z_i^{\mathrm{nc}-} + Z_i^{\mathrm{nc}+} Z_i^{\mathrm{nc}-}}{Z q_i}. \tag{S11}$$

Note that this is divided by q_i to account for double counting of the state in which only site i is bound. This calculation only allows pairs of sites to bind cooperatively and does not allow polymerization of bound factors across DNA.

While calculations that explicitly calculate the weight of every binding state scale in $O(2^n)$, the dynamic algorithm presented here can calculate occupancy in linear time, given that the binding sites are already sorted and there is a finite maximum distance at which cooperativity of binding can occur.

Action at a Distance Transcription factors bound to DNA interact according to the distance between sites, where nearby sites have strong interactions and distant sites do not interact. In order to define rules governing these interactions, we define a function that determines the efficiency of interaction between sites i and k. We report the the distance between sites as

$$d(i,k) = \min\left(\left|m_i - n_k\right|, \left|n_i - m_k\right|\right), \tag{S12}$$

and the efficiency of interaction is given by

$$g(i,k,A,B) = \begin{cases} 1 & d(i,k) \leq A \\ 1 - \frac{d(i,k)-A}{B} & A < d(i,k) < A+B \\ 0 & A+B \leq d(i,k) \end{cases}, \tag{S13}$$

where A and B govern the shape of this interaction.

Coactivation We allow bound factors to take one of two possible states: an activating state f^A and a quenching state f^Q. For obligate repressors $f^Q_{i[m,n;a]} = f_{i[m,n;a]}$, and similarly for obligate activators $f^A_{i[m,n;a]} = f_{i[m,n;a]}$. For proteins that can take on both possible states we allow state switching from a default state to an induced state based on the occupancy of nearby inducing factor. This is the case for the transcription factor Hunchback, a quencher that can be induced to activate by the presence of bound Bicoid or Caudal. For sites i and k, we define a function that returns the efficiency of this interaction based on the distance between these two sites

$$f^Q_{i[m_i,n_i;a_i]} = f_{i[m_i,n_i;a_i]} \prod_k \left(1 - g(i,k,D_c,50) E^C_{a_k} f_{k[m_k,n_k;a_k]}\right), \tag{S14}$$

$$f^A_i = f_i - f^Q_i \tag{S15}$$

where D_c is a free parameter giving the maximum distance at which coactivation is 100% efficient and $E^C_{a_k}$ is a free parameter giving the maximum efficiency with which factor a_k induces activation of factor a_i. This product occurs over all k binding sites within the locus.

Quenching Bound repressors quench the activity of bound activators. This results in an an effective occupancy of each each activator, F, which is given by

$$F_i = f^A_i \prod_k \left(1 - g(i,k,100,50) E^Q_{a_k} f^Q_k\right), \tag{S16}$$

where $E^Q_{a_k}$ is a free parameter giving the efficiency with which factor a_k quenches. This product occurs over all k binding sites within the locus.

Summation of Recruited Transcriptional Adaptors The remaining bound, unquenched activators are free to recruit transcriptional adaptors that enhance interaction with the promoter. The number of adaptors recruited to a sequence bounded by bases p and q is given

by

$$N_{[p,q]} = \sum_k F_k E^A_{a_k} I(k,p,q), \tag{S17}$$

where the $E^A_{a_k}$ is the activating efficiency of factor a_k and $I(k,p,q)$ is a function that specifies whether site k falls between p and q, given by

$$I(k,p,q) = \begin{cases} 1 & m_k \geq p, n_k < q \\ 0 & \text{Otherwise} \end{cases} \tag{S18}$$

Three State Transcription Model We allow a promoter to have three states: (1) an open state with no polymerase bound, (2) a paused, bound state, and (3) an actively elongating state.

$$\text{Open} \underset{k_{-1}}{\overset{k_1}{\rightleftharpoons}} \text{Bound} \overset{k_2}{\rightarrow} \text{Elongating} \overset{k_3}{\rightarrow} \text{Open}. \tag{S19}$$

The probability of the system being in any state is given by the system of differential equations

$$\begin{aligned} \frac{dP_1}{dt} &= -k_1P_1 + k_{-1}P_2 + k_3P_3 \\ \frac{dP_2}{dt} &= k_1P_1 - (k_{-1} + k_2)P_2 \\ \frac{dP_3}{dt} &= k_2P_2 - k_3P_3. \end{aligned} \tag{S20}$$

At steady state, the probability of each state is given by

$$\begin{aligned} \bar{P}_1 &= \frac{k_3(k_{-1} + k_2)}{q} \\ \bar{P}_2 &= \frac{k_1k_3}{q} \\ \bar{P}_3 &= \frac{k_1k_2}{q}, \end{aligned} \tag{S21}$$

where $q = k_3(k_{-1} + k_2) + k_1(k_2 + k_3)$. The rate of transcription is the rate at which the

system moves through state 3

$$\bar{P}_2 k_2 = \bar{P}_3 k_3 = \frac{k_1 k_2 k_3}{k_3 k_{-1} + k_3 k_2 + k_1 k_2 + k_1 k_3}. \tag{S22}$$

Given that we are modeling a developmental promoter displaying paused polymerase, we assume that the rate limiting step is initiation. Formally, we assume that as $k_2 \to \infty$, the rate of transcription goes to some value $R_{\max}$. Observing the boundary behavior of eqn. S22, we find that

$$k_1 k_3 = R_{\max}(k_1 + k_3). \tag{S23}$$

Substituting this relation into eqn. S22 we find the transcription rate is given by

$$R = \frac{k_2}{1 + K_d + \frac{k_2}{R_{\max}}}, \tag{S24}$$

where $K_d = \frac{k_{-1}}{k_1}$. We treat the reaction rate k_2 as a rate catalyzed by adaptor factors recruited to the promoter by enhancers, where each recruited adaptor gives a linear reduction in the energy barrier to transcription initiation. This rate is given by

$$k_2 = c \exp\left(\frac{-\Delta A}{RT}\right), \tag{S25}$$

and

$$\Delta A = \theta - N \tag{S26}$$

where θ is the barrier to initiation and N is the reduction $\Delta\Delta A$ in the activation energy. Then the rate of transcription can be expressed as

$$R = R_{\max} \frac{c \exp(\frac{N-\theta}{RT})}{R_{\max}(1 + K_d) + c \exp(\frac{N-\theta}{RT})}.$$

Simplifying, we get

$$R = \frac{R_{\max}}{1 + \exp\left(\frac{\theta - N}{RT} + \ln(\frac{R_{\max}(1+K_d)}{c})\right)}.$$

Because the quantity θ is a free parameter it can simultaneously account for the energy barrier of initiation as well as the quantity $\ln(\frac{R_{\max}(1+K_d)}{c})$. Additionally, the scale of θ and N are set by free parameters so they can also adjust for RT. For these reasons, we simply use the function

$$R = \frac{R_{\max}}{1 + \exp(\theta - N)}. \quad \text{(S27)}$$

Sequences

The PCR primers used in cloning are below.

`5′ Extention Primer` TGGGTTTTATTAACTTACATACATACTAGAATTCGAGCTCGC-CCGGGGATC

`3′ Extention Primer` GTTGTTGACTGTGCGGCGGTCACAGCTCGAGTGTGCT-GCTCTCAGCCACCCCGCGCCCTTTTATACCGCTGCGCTC

References

[1] Berg G, von Hippel PH. Selection of DNA binding sites by regulatory proteins. Statistical-mechanical theory and application to operators and promoters. Journal of Molecular Biology. 1987;193:723–50.

[2] Gregor T, Wieschaus EF, McGregor AP, Bialek W, Tank DW. Stability and nuclear dynamics of the Bicoid morphogen gradient. Cell. 2007;130:141–152.

[3] Maerkl SJ, Quake SR. A systems approach to measuring the binding energy landscapes of transcription factors. Science. 2007;315:233–237.
