# Supplementary figures and images for: Correction: A sequence level model of an intact locus predicts the location and function of nonadditive enhancers
Source: PLoS One. 2018 May 7;13(5):e0197211. doi: 10.1371/journal.pone.0197211 (PMC5937780; doi:10.1371/journal.pone.0197211)

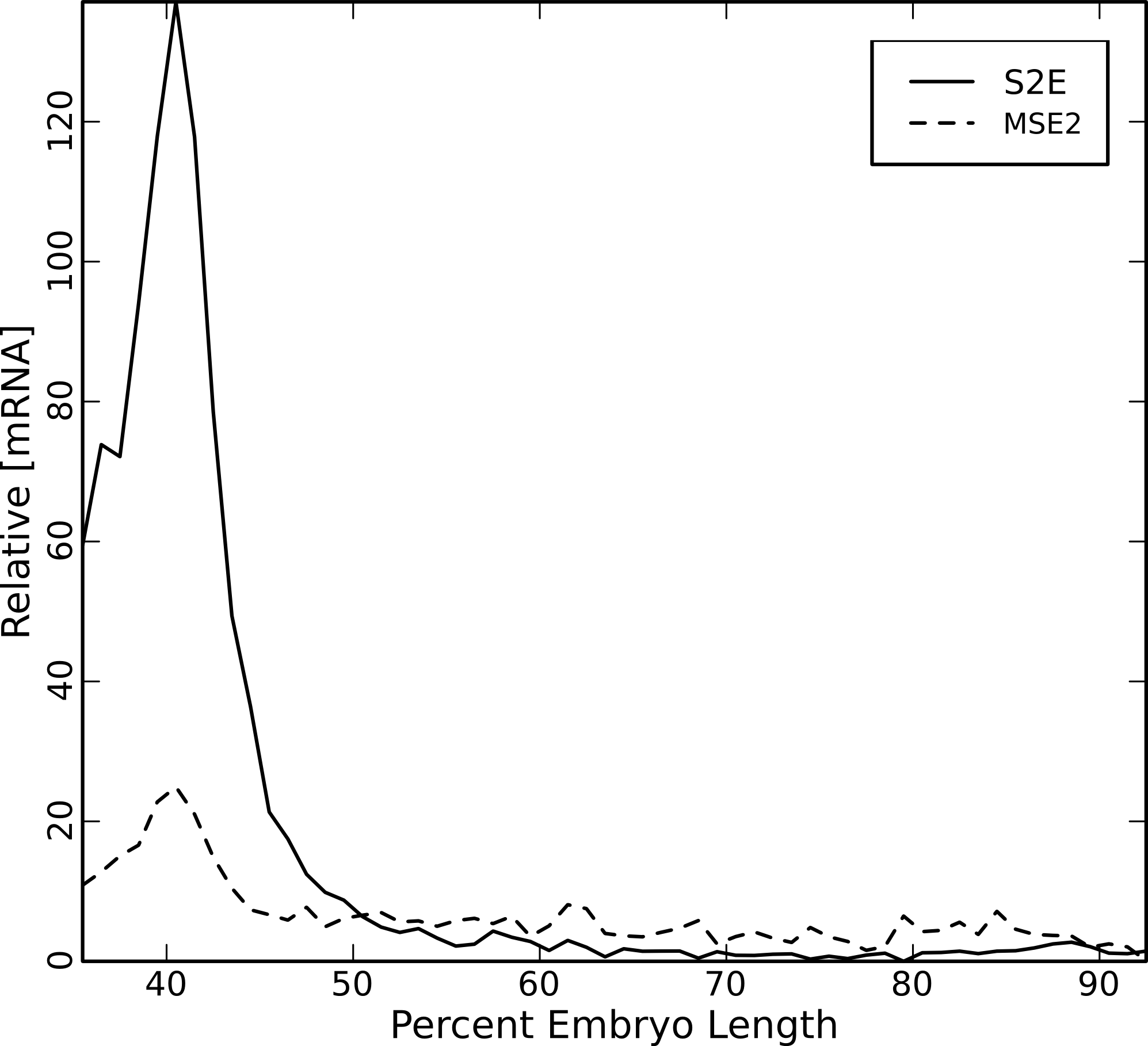

Supplement: S2 Fig — The 480 bp MSE2 fragment and the 698 bp S2E (dm3 coordinates 2R:5865217–5865913) were placed upstream of lacZ and cloned into the AttP2 site in Drosophila. Mean fluorescent in-situ hybridization (FISH) intensity at nuclear cycle 14 timepoint 6 is reported with S2E in solid lines and MSE2 in dashed lines. 15 embryos containing S2E were imaged, giving between 47 and 63 nuclei per AP position. 8 embryos containing MSE2 were imaged, giving between 26 and 37 nuclei per AP position. Peak expression of S2E is 5.5 times greater than that of MSE2, despite only containing 218 additional bases. (TIF) [file pone.0197211.s001.tif]
